# Radical collaboration during a global health emergency: development of the RDA COVID-19 data sharing recommendations and guidelines

**DOI:** 10.12688/openreseurope.13369.1

**Published:** 2021-06-16

**Authors:** Brian Pickering, Timea Biro, Claire C. Austin, Alexander Bernier, Louise Bezuidenhout, Carlos Casorrán, Francis P. Crawley, Romain David, Claudia Engelhardt, Geta Mitrea, Ingvill Constanze Mochmann, Rajini Nagrani, Mary O'Brien-Uhlmansiek, Simon Parker, Minglu Wang, Leyla Jael Castro, Zoe Cournia, Kheeran Dharmawardena, Gayo Diallo, Ingrid Dillo, Alejandra Gonzalez-Beltran, Anupama Gururaj, Sridhar Gutam, Natalie Harrower, Jitendra Jonnagaddala, Katherine McNeill, Daniel Mietchen, Amy Pienta, Panayiota Polydoratou, Marcos Roberto Tovani-Palone

**Affiliations:** 1 University of Southampton, Southampton, UK; 2 Digital Repository of Ireland / Royal Irish Academy, Dublin, Ireland; 3 Environment and Climate Change Canada, Canada, Canada; 4 Centre of Genomics and Policy / McGill University, Montreal, Canada; 5 University of Oxford, Oxford, UK; 6 European Commission, Brussels, Belgium; 7 Good Clinical Practice Alliance - Europe (GCPA) / Strategic Initiative for Developing Capacity in Ethical Review (SIDCER), Leuven, Belgium; 8 European Research Infrastructure on Highly Pathogenic Agents, Paris, France; 9 Göttingen State and University Library, Göttingen, Germany; 10 Carol I National Defence University, Bucharest, Romania; 11 GESIS-Leibniz Institute for the Social Sciences, Cologne, Germany; 12 Leibniz Institute for Prevention Research and Epidemiology (BIPS), Bremen, Germany; 13 Research Data Alliance, St. Louis, USA; 14 The German Cancer Research Center (Deutsches Krebsforschungszentrum, DKFZ), Heidelberg, Germany; 15 York University, Toronto, Canada; 16 ZB MED Information Centre for Life Sciences, Köln,, Germany; 17 Biomedical Research Foundation, Academy of Athens, Athens, Greece; 18 Cytrax consulting, Canberra, Australia; 19 Université Bordeaux, Bordeaux, France; 20 Data Archiving and Networked Services (DANS), The Hague, The Netherlands; 21 Science and Technology Facilities Council, UK Research & Innovation, Oxford, UK; 22 National Institute of Allergy and Infectious Disease, National Institutes of Health, Fairfax, USA; 23 ICAR-Indian Institute of Horticultural Research, Bengaluru, India; 24 University of New South Wales / School of Population Health, Kensington, Australia; 25 Harvard University, Cambridge, USA; 26 School of Data Science, University of Virginia, Charlottesville, USA; 27 ICPSR-University of Michigan, Ann Arbor, USA; 28 International Hellenic University, Marseille, France; 29 University of São Paulo, São Paulo, Brazil

**Keywords:** COVID-19, global health, public health, pandemic, epidemic, data sharing, radical collaboration, Research Data Alliance

## Abstract

**Background:** The coronavirus disease 2019 (COVID-19) global pandemic required a rapid and effective response. This included ethical and legally appropriate sharing of data. The European Commission (EC) called upon the Research Data Alliance (RDA) to recruit experts worldwide to quickly develop recommendations and guidelines for COVID-related data sharing.

**Purpose: **The purpose of the present work was to explore how the RDA succeeded in engaging the participation of its community of scientists in a rapid response to the EC request.

**Methods:** A survey questionnaire was developed and distributed among RDA COVID-19 work group members. A mixed-methods approach was used for analysis of the survey data.

**Results:** The three constructs of radical collaboration (inclusiveness, distributed digital practices, productive and sustainable collaboration) were found to be well supported in both the quantitative and qualitative analyses of the survey data. Other social factors, such as motivation and group identity were also found to be important to the success of this extreme collaborative effort.

**Conclusions:** Recommendations and suggestions for future work were formulated for consideration by the RDA to strengthen effective expert collaboration and interdisciplinary efforts.

## Disclaimer

All views and opinions expressed are those of the co-authors, and do not necessarily reflect the official policy or position of their respective employers, or of any government, agency, or organization.

## 1. Introduction

As the coronavirus disease 2019 (COVID-19) epidemic began to develop from a public health emergency in China into a global threat, Xie *et al.* (2020) compared the growing pandemic with a global information crisis. They called upon the international community to collaborate, share data and information to address the developing global health emergency. Their call to action uses the definition by Koplan *et al.* (2009) that distinguishes global health from the more narrowly defined concepts of public health and international health:

“… global health is an area for study, research, and practice that places a priority on improving health and achieving equity in health for all people worldwide. Global health emphasises transnational health issues, determinants, and solutions; involves many disciplines within and beyond the health sciences and promotes interdisciplinary collaboration; and is a synthesis of population-based prevention with individual-level clinical care.” ( Koplan *et al.*, 2009, p.1995).

Picking up on this definition, Beaglehole & Bonita (2010, p. 1) shortened it, making it more action-oriented and emphasising the critical need for collaboration:

“… global health is *collaborative trans-national research and action for promoting health for all*.”

Such collaboration, however, especially as nation after nation imposed lockdowns and restricted travel, called for an appropriate virtual environment to connect expertise from across the globe. Given the imperative of a global health emergency, generating appropriate cross-disciplinary guidance requires intensive effort across areas of expertise within a pressured timeframe. This necessitates collaboration of a different order than is usually the case. The success of such an environment in leading to data and information sharing outcomes depends on individual experts’ willingness to engage and maintain sustained effort. Collaborators need to be motivated if they are to use an appropriate environment to work together from day one, adapting their usual engagement practices. The present paper explores the two-fold challenge for successful collaboration at times of global health emergencies: the organisational processes to encourage engagement and the associated response of individuals to commit to it.

### 1.1 Background to the COVID-19 Research Data Alliance (RDA) working group

Judicious and transparent data sharing is crucial to the research and development process that leads to outbreak modelling, diagnostics, therapeutics, and vaccines to prepare for and respond to epidemics. It also enables global and public health professionals, social scientists, and policy-makers – from local communities to international organisations – to make informed decisions, establish policies to contain disease spread, mitigate the consequences of an outbreak, and save lives.

The present study explores how experts were engaged to work collaboratively in response to an immediate global health threat and provide a cooperative framework for the development of the *Research Data Alliance (RDA) COVID-19 Recommendations and Guidelines on Data Sharing* ( RDA COVID-19 WG, 2020a). Understanding how this process functioned provides valuable insights that may inform collaborative responses to future global health or other large-scale emergencies. These insights provide lessons for collaborating globally, but also for working together across disciplines, cultures, sectors, and jurisdictions in order to realise an adequate and measured response.

### 1.2 Development of COVID-19 data sharing recommendations and guidelines

The World Health Organisation’s (WHO) statement on data sharing during public health emergencies foretold the critical need for the timely sharing of relevant information during an emerging pandemic. On 28 May 2020, the G7 Science and Technology Ministers’ Declaration on COVID-19 was issued, calling for government-sponsored COVID-19 epidemiological and related research data and information, along with scientific results, to be made accessible to the public to the greatest extent possible. At the same time, there was strong support for recognising open research data as a key component of pandemic preparedness and response, evidenced by the 117 cross-sectoral signatories to the Wellcome Trust’s update of a previous statement on data sharing in public health emergencies (published on 31 January 2020) and the agreement by 30 leading publishers on immediate open access to COVID-19 publications and their underlying data. 

The global community’s response was punctual, initially through efforts that were largely independent of one another, but with an eye to convergence. For instance, a number of data visualisation web platforms demonstrating cases of infections and deaths by country were launched at the start of the pandemic to convey the strategic importance of standardised data sharing ( Arora *et al.*, 2021; Dong *et al.*, 2020). See, also, the Austin *et al.* (2020a PREPRINT) survey of COVID-19 data sources, models, and visualisations.

The European Commission (EC) was also keen to seek coordination and alignment across its member states, and to identify ways to leverage synergies aimed at ensuring an efficient response to the pandemic. The EC’s Directorate-General for Research and Innovation drafted an action plan, developed through dialogues with other EC Directorates-General, and publicly endorsed by the EU research and innovation ministers on 7 April 2020, establishing ‘10 priority actions for coordinated research and innovation actions’. Action #9 was the establishment of a European data sharing platform for severe acute respiratory syndrome coronavirus 2 (SARS-CoV-2) and coronavirus-related information exchange. The EC, together with the European Bioinformatics Institute of the European Molecular Biology Laboratory (EMBL-EBI), the European Infrastructure for Life Sciences (ELIXIR), and other partners, came together to deliver the European COVID-19 Data Platform, an important contribution to the European Open Science Cloud (EOSC).

Overall, these actions aimed at aligning and exploiting activities that could speed up and improve the storage of, access to, and sharing of research data and metadata on SARS-CoV-2 and the COVID-19 disease that it causes. The objective was to foster the rapid, open, and efficient sharing of relevant research data and metadata across member states and internationally, while also implementing the FAIR ( **F** indable, **A** ccessible, **I** nteroperable, and **R** eusable) data principles and fully respecting the European General Data Protection Regulation (GDPR) ( Wilkinson *et al.*, 2016). These actions served to support global health surveillance, accelerate scientific discovery, and facilitate the speedy provision of sound evidence for effective policy making.

To rapidly advance best practices related to data management, governance, and sharing, the EC approached the RDA on 19 March 2020 to seek the creation of a fast-tracked, emergency COVID-19 data sharing working group (WG). The WG would (a) build upon and expand already existing data sharing principles for use during public health emergencies and (b) leverage the global RDA forum and processes for the fast development of recommendations and guidelines for data sharing during the COVID-19 pandemic.

As the work of the RDA group proceeded, it became apparent that the recommendations and guidelines would need to address specificities related to omics and clinical research as well as the management and sharing of epidemiological and social sciences data. The scope of the work was progressively broadened by the RDA to include community participation, legal and ethical challenges, data involving Indigenous populations, research software, and computer coding.

### 1.3 The RDA and the COVID-19 Working Group

The RDA is a non-profit, community-driven, international organisation established in 2013 that brings together more than 11,000 members from 145 countries. It “ provides a neutral space where members can come together to develop and adopt infrastructure that promotes data-sharing and data-driven research and accelerate the growth of a cohesive data community that integrates contributors across domain, research, national, geographical and generational boundaries”. Approximately 23% of RDA members are researchers ( Chan, 2019), representing roughly 0.05% of OECD researchers ( OECD, 2016). Chan (2019) defines researchers as, "primary research data producers and users," while the OECD definition is, "professionals engaged in the conception and creation of new knowledge, products, processes, methods and systems, as well as those directly involved in the management of the projects concerned."

The RDA Foundation is a not-for-profit charitable global organisation established in the UK responsible for RDA operations. Its total annual income is £229,200 (2018), corresponding to approximately 5% of the RDA’s total annual budget. The RDA is financed by funders (Australian Government Department of Education and Training; European Commission; US National Institute of Standards and Technology; US National Science Foundation; French Ministry of Higher Education, Research and Innovation), other financial supporters (Alfred P. Sloan Foundation, JISC, MacArthur Foundation, and Wellcome Trust), in-kind contributions (staff and personnel support; hosting international plenaries), and organisational membership fees. More details on the legal entity and its finances are available at https://www.rd-alliance.org/about-rda/rda-foundation. 

The RDA promotes an inclusive approach to data challenges covering the complete data lifecycle and engaging data producers, users, and stewards. RDA membership subscribes to the following guiding principles: openness, consensus, inclusiveness, harmonization, community driven, and non-profit technology neutral.

Organisations typically function within some level of hierarchical or flat internal structure, depending on the number of management layers ( Handel, 2014; Lee & Edmondson, 2017; Wulf, 2012). Having no middle management at all, the RDA is an extreme example of a flat organisational structure. It provides a framework for self-managed volunteer groups to achieve defined objectives:

**Communities of practice (CoP):** Specific discipline/research domain focused groups that provide the opportunity to investigate, discuss, coordinate and provide knowledge to discuss data-related trends and challenges and collaborate on implementing solutions. CoP is a recent addition.
**Interest groups (IGs):** Thematically focused groups that are typically long-lasting; members germinate new Working Groups to tackle data-sharing challenges.
**Working groups (WGs):** Task focused groups with a finite lifespan that work towards a specific outcome or solution to a data-sharing challenge.

The following organisational bodies support the work of the volunteer groups:

**Technical advisory board (TAB):** Provides technical expertise and advice to the Council, and assists in development, review and promotion of the CoP, IGs, and WGs.
**Leadership council:** Maintains the vision of RDA, ensuring the guiding principles of the organisation are maintained, and formally endorses RDA working and interest groups and recommendations.
**Organisational advisory board:** Represents the interests of organisational members, ensuring that their input and needs play a role in guiding the programmes and activities of the RDA.
**Secretary general/chief executive officer:** Leadership of RDA’s membership, effective management of the RDA organisation, engagement with RDA stakeholders and organisations, and sustainable stewardship of a dynamic, active, and high-impact community.
**Secretariat:** Responsible for the administration and daily operations of RDA.

Normally, RDA groups are a grassroots initiative. The case of the RDA COVID-19 WG was unusual in that it was created at the request of the EC, one of the RDA funders. The flat organisational structure of the RDA, unencumbered by hierarchical and bureaucratic processes, provided a framework for rapid response and engagement of a global community of volunteers committed to sharing their expertise.

**Figure 1.**
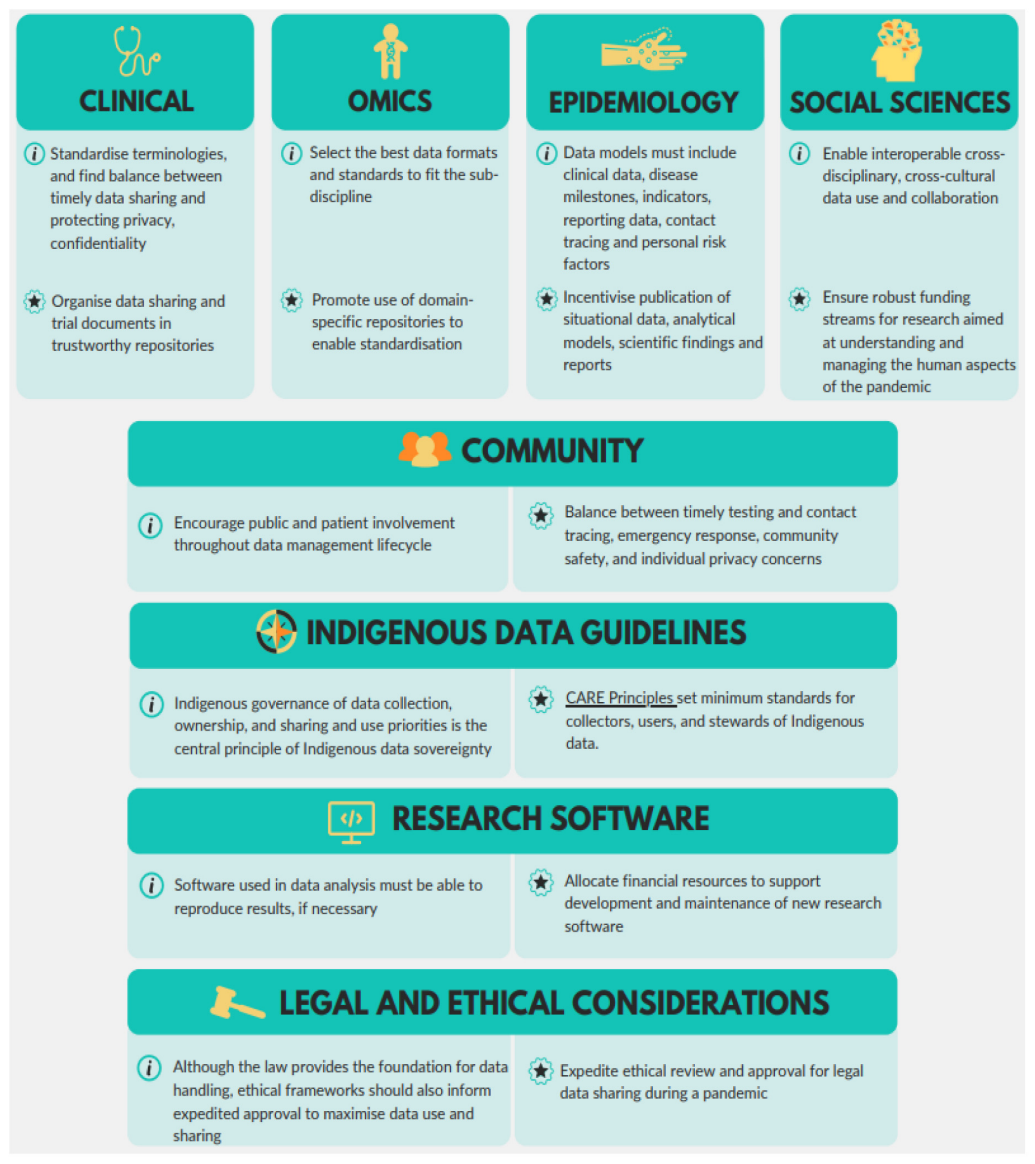
A collaborative cross-disciplinary effort – Schematic representation of the RDA COVID-19 Working Group and eight sub-working groups. [SOURCE: RDA COVID-19 WG (2020c), with permission].

The RDA COVID-19 WG brought together members with cross-disciplinary global expertise. Six hundred volunteers joined the WG to produce a set of data sharing recommendations and guidelines for the COVID-19 pandemic over a 3-month period. Eight sub-working groups were set up to handle four domain-specific research areas of clinical, omics, epidemiological, and social science data sharing, with four cross-cutting areas of Indigenous peoples, community participation, research software, and legal & ethical issues ( Figure 1). References for all sub-WG contributions, supporting outputs, and related publications were managed in a single database that continues to be updated, including for the present paper ( RDA COVID-19 WG, 2020b). This framework enabled members to focus the conversations and provide an initial set of recommendations and guidelines in a tight timeframe over only three weeks. One hundred and sixty-four of the volunteers (ca. 27% of the original 600) worked directly on the recommendations and guidelines. They came together to assess how data from multiple disciplines could inform responses to a pandemic and to formulate their findings on sharing data, computer code, contextual information, and other research outputs under the present COVID-19 circumstances.

### 1.4 The novelty and impact of the outputs

The RDA COVID-19 (2020a) recommendations and guidelines aimed at outlining a set of best practices for data sharing in a global health emergency to support scientific research and policymaking, including an overarching frame of reference, common tools and processes, and principles that can be embedded into research practice. This set of best practices was developed within a global framework during a pandemic. Underlying this framework, however, is a process we describe as ‘radical collaboration’, which provides a model framework for rapid organisation and output, in this case during global health emergencies, regardless of their geographical scale.

The recommendations and guidelines address general aspects of data practice and the adoption of research-domain community standards, including FAIR data principles, TRUST ( **T** ransparency, **R** esponsibility, **U** ser focus, **S** ustainability and **T** echnology) principles, and CARE ( **C** ollective benefit, **A** uthority to control, **R** esponsibility, and **E** thics) principles with respect to Indigenous data. ( Lin *et al.*, 2020; RDA COVID-19 WG, 2020a). They provide overarching recommendations as well as actionable guidelines – aiming to respond to the needs of a range of stakeholders: from policy makers and funders to infrastructure managers, researchers, and data practitioners. The guidelines detail disciplinary practice while discerning the foundational overarching challenges and recommendations that appeared across the four research topics and the cross-cutting themes. Austin *et al.* (2020b PREPRINT) have summarised key points of the detailed 143-page report ( RDA COVID-19 WG, 2020a), highlighting the relevant findings, shining a spotlight on the process, and suggesting how these developments can be leveraged by the wider scientific community.

In carrying out this work, it became clear that the nature of rapid, effective collaboration between experts from multiple geographies and disciplines made this an exceptional exercise that benefited from leveraging the RDA structures and procedures. The focus from the beginning was placed on balancing both timeliness and data quality. At the same time, and as developed in the study described below, the ultimate success of this initiative required the dedicated effort of many volunteer experts working together – in many cases for the first time, and in novel ways dictated by the exceptional circumstances.

Given the effective and rapid development of the recommendations and guidelines, this paper seeks to examine the key characteristics that enabled this endeavour and led to its success, so that other collaborations may learn and follow suit. The following sections describe how the constructs of radical collaboration found in the literature were operationalised via a survey. The survey comprised both open and closed questions to identify the demographic characteristics of participating experts and their perceptions of the experience.

## 2. Theoretical background

To analyse key aspects of the RDA COVID-19 working group initiative and experience, we draw upon a set of increasingly accepted and expanding theories and concepts, covering radical collaboration and intergroup processes of defensiveness, as a way of dealing with common fears about personal significance and competence in a collaborative environment.

### 2.1 Radical collaboration

Since the foundation of the RDA, reflections and research about it as an innovative and influential organisation tackling research data management issues have come primarily from library research literature. Shortly after the launch of the RDA, *D-Lib Magazine* published a special issue guest editorial by two RDA Council members and the secretary-general ( Berman *et al.*, 2014). This special issue featured articles describing several RDA groups and their activities as well as a status report on the RDA organisational structure ( Parsons, 2014).

Four years later, a special issue of the *Research Library Issues* ( Ruttenberg & Waraksa, 2018) focused on the collaboration thesis of research data management communities. In this context, radical collaboration was introduced as an operational framework by McGovern (2018b), based on Scott's (2017) earlier discussion of radical candor which was later updated ( Scott, 2019). Radical collaboration, however, was already known in the context of conflict resolution and mediation ( Tamm & Luyet, 2010). In this same special issue, Nurnberger (2018) applied the concept of ‘radical collaboration’ to her own experience with RDA groups’ formation and maintenance, and proposed recommendations for developing sustainable institutional research data management services. In the present study, we applied and tested radical collaboration to the RDA COVID-19 WG activities after the recommendations and guidelines had been produced and the main work was completed.

McGovern (2018a, p.6) states:

“The concept of radical collaboration means coming together across disparate, but engaged, domains in ways that are often unfamiliar or possibly uncomfortable to member organizations and individuals in order to identify and solve problems together, to achieve more together than we could separately.”

McGovern further develops three working concepts to help analyse the process of a radical collaboration: inclusive community, distributed digital practices, and productive and sustainable collaboration. We made use of this theoretical framework in our study and operationalised it into a series of survey questions, to gather participants’ perceptions of this collaborative process and describe the diverse aspects of the RDA COVID-19 WG experiences.

### 2.2 Dealing with defensiveness

In this paper radical collaboration is discussed as a framework for the description and implementation of effective cooperation between an otherwise disparate group of individuals ( McGovern, 2018a). However, given that individuals would come from different disciplines and cultures, defensiveness could become a specific challenge in this context ( Tamm & Luyet, 2010). Through surveying RDA COVID-19 WG members and collecting data on individuals’ experiences, perceptions, and thoughts about the working process, we had an opportunity to also shed light on a deeper level of the intergroup processes ( Hornsey & Imani, 2004). Especially in the online environment, that is in the *distributed digital practices* of radical collaboration, perceptions of group affiliation influence anxiety levels and a willingness to engage and work together ( Amichai-Hamburger, 2012). This applies to the RDA COVID-19 WG. Individual members would need to quickly drop their guard and unitary disciplinary focus (related to defensiveness) to be prepared to collaborate with people they may never have met before and whom they may not understand in disciplinary terms. Effective collaboration would require the suspension of individual defensiveness for the common (external) motivation of getting the job done. The RDA COVID-19 WG initiative also brought together new RDA members who may have had their issues around defensiveness related to being newcomers, and existing RDA members who may have presented an attitude of ‘this is how we’ve always done it,’ leading in turn to yet more defensiveness.

### 2.3 The research question

A key motivation behind the present paper derives from the novelty of the initiative. As discussed above, the WG came together in a very short time. It adopted a model for collaboration and communication based on existing RDA procedures but tailored to the specific context and employing writing sprints, various teams with targeted tasks, and other collaborative methods to meet the short timeframe for development. The following question guided the present investigation:

How did the RDA successfully engage the participation of its community of scientists in a rapid response to develop data sharing recommendations and guidelines during the global challenge of COVID-19?

The primary focus of the study is radical collaboration, addressing aspects related to the novelty of the joint work and questions of defensiveness. However, it is also important to consider how the motivation of volunteer experts is sustained through the lenses of the experienced challenges and rewards.

## 3. Methods

### 3.1 Ethics

The research ethics literature highlights the need to respect participants. Most institutional research boards (IRBs) reference the Belmont report implicitly or explicitly, and require respect for the research participant, the avoidance of harm (benevolence/non-malevolence), and equanimity of treatment. See, also, ALLEA (2017).

In practice, respect begins with participant consent. They need to be given sufficient information to be able to decide freely whether to take part. For the present study, some care was needed so that participants did not feel that their ongoing RDA membership and their participation were interdependent. With that in mind, a request was sent to all RDA COVID-10 WG members stressing that their participation was entirely voluntary. This was further emphasised in the introductory text explaining the purposes of the survey.

Responding to the request to participate would include both long-term RDA members and those who had specifically joined for the RDA COVID-19 initiative. One way to avoid harm (non-malevolence) and promote equanimity would be to ensure that responses could not be linked to specific members and to make no distinction based on length of RDA membership. In addition, responses should be pseudonymised to remove any identifiers such as IP Address or participant RDA login details.

Identifying individuals may also affect participant responses. If they felt they could not be completely open about their experience, it is possible they may be concerned that this would affect their RDA membership in future.

With all of this in mind, once the survey questionnaire was finalised, the research protocol and proposed analysis methods were submitted to and approved by the University of Southampton, Faculty of Engineering and Physical Sciences in the UK (ref.: ERGO/FEPS/61523.A1). The University research ethics policy is publicly accessible. In addition, the pseudonymised results were then anonymised using sdcMicro ( Templ *et al.*, 2015) to further reduce re-identification risk.

### 3.2 Design of the survey questionnaire

The design of the survey questionnaire (see *Extended data,* David *et al.*, 2021c) was based on the three main dimensions of radical collaboration ( McGovern, 2018a):

- inclusive community;
- distributed digital practices; and
- productive and sustainable collaboration.

The objective was to gather members’ experiences via a mixture of Likert-scale responses and open-ended questions. The survey gathered respondents’ demographic characteristics and information regarding their participation in the RDA COVID-19 WG. For instance, how they had learned about the initiative (A-8) and what motivated them to join the WG (A-7), whether they had observed, contributed to, chaired or co-moderated a subgroup (A-9), how they had participated (A-10), and so forth.

***Radical collaboration indicators***

**An inclusive community**

In discussing the radical collaborative community, McGovern (2018a, p.14) emphasised that inclusiveness should go beyond social and demographic aspects. Professional career stage and technical inclusion are equally important in order for a diverse group of community participants to be challenged and learn from the many varied points of view not previously familiar to them. McGovern’s optimism echoes the importance of ‘weak ties’ for innovation previously discussed in the literature. Bringing together experts from diverse fields, weakly tied, unlikely to have collaborated before, generates a network of expertise built on novel associations deriving from the need to work quickly ( Friedkin, 1980; Granovetter, 1973).

The radical collaboration construct of inclusive community was operationalised in the questionnaire by the following measures:

**Socio-demographics** (Question A1-A6)
• A-1: Geographic Inclusion – residence of WG members (Africa, Americas, Asia, Europe, and Oceania)
• A-2: Scientific Domain Inclusion – domains of research or expertise among WG members (7 domains)
• A-3: Occupational inclusion – roles or positions of WG members (10 categories)
• A-4: Organisational inclusion – organisation types of WG members’ workplace (7 categories)
• A-5: Social inclusion – career stages of WG members (earlier and advanced stages)
• A-6: Newcomer inclusion – whether the WG members had been RDA members before
**Perceptions of inclusion** (Question A-11)
• Social inclusion (e.g. common goals and shared problems, low resources settings involvement)
• Demographic inclusion (e.g. geographical provenance, education, organisational affiliation)
• Professional inclusion (e.g. professional roles/positions, domain and area of expertise)
• Career stage inclusion (e.g. support/inclusion for early careers, and other)
• Newcomers inclusion (e.g. support for newcomers, induction/onboarding to WG and RDA)
• Technical inclusion (e.g. technical base used, its availability and adoption, level of skill necessary)
• Language inclusion (Use of language, terms etc)

**Distributed digital practices**

The construct of ‘distributed digital practices’ examines aspects of digital working and of the collaborative environment ( McGovern, 2018a, p.16–18). As McGovern observes, digital practices are moving from an individually or collectively controlled process towards a more distributed environment where more domain experts come together from disparate backgrounds and with no other common connection beyond the internet-mediated forum of those sharing similar interests. Such a virtual environment can be very challenging, especially in a diverse community whose members represent varying levels of technical adeptness. A successful radical collaboration can still thrive in such an environment through a cumulative and iterative process. It becomes responsive to change, building on past experience and supported by coordinated processes and structures.

In the case of the RDA COVID-19 WG, all tasks were distributed or, more accurately, volunteered for by members and organised at the study group (sub-working group) level. Each member voluntarily assumed a work role (some chose different roles such as contributor or member at different times and for different sections of the study). Given that team members met and worked from home offices, the only elements that kept them connected were efficient online communications and platforms such as Zoom ^TM^, Google Drive ^TM^, and Zotero ^TM^.

Respondents were asked to rate the success of the RDA COVID-19 WG collaboration as (Question B-1):

- Cumulative process (e.g. progressively increase the knowledge and quality of the recommendations)
- Reiterative process (e.g. following various rounds of analysis, discussion and writing)
- Responsive to change and particular context (e.g. adapted to the changes in work environments and digital transition caused by COVID-19)
- Leveraging lessons from the past (e.g. bringing forward previous collaborative experiences and lessons learnt to improve and speed up the process)
- Learning process (e.g. has led members and community to acquire new information, understanding and skills)
- Contributive process (e.g. leveraging contributions from individual members and groups)
- Consensus based process (e.g. developments and decisions were discussed and accepted by all)
- Non-hierarchical/bottom-up process (e.g. giving voice and opportunity to all to shape the direction of the initiative)
- Coordinated process/providing necessary support structures (e.g. RDA structures were in place to provide coordination and support: chairs, moderators, review processes, secretariat)
- Goal clarity (e.g. project purpose, timeline, and deliverables)
- Division of labour (e.g. assignment and clarity of responsibilities)

**Collaborative engagement and commitment**

Members were asked what their motivation was for joining the COVID-19 WG. Deci & Ryan (2000) distinguish between intrinsic and extrinsic motivation. Extrinsic motivation relates to undertaking the activity to achieve a tangible outcome. It would include factors such as having some impact on the course of the COVID-19 pandemic, or professional benefit for academics who may need to demonstrate external connections and collaboration. Such extrinsic motivation tends to be short-lived and situation-dependent: once the RDA COVID-19 recommendations and guidelines were agreed and published, the extrinsic motivation would be expected to disappear. Intrinsic motivation relates to inherently important factors for the individual. This might include, for instance, prosociality or eagerness to learn. Intrinsic motivations are more resilient and more persistent. In the case of intrinsically motivated individuals, therefore, they might continue to collaborate and offer time and effort, even after the initial stimulus (producing the recommendations) had passed. The survey asked members to answer the following question:

- A-7: What motivates members to get involved? Choose from a list of possible motivations ranging from individual development and willing to share, shared challenges, to belief in data sharing and collaboration, and commitment to RDA vision.

McGovern (2018a) emphasized that a radical collaboration is not only inclusive in terms of its members’ diverse backgrounds nor sustained by individual members’ motivations. It needs communal commitment and interactive engagement from all group members ( McGovern, 2018a, p.11). Members of such collaborations will be challenged by different perspectives than their own, and the ideal of this community practice cannot be achieved until members become deeply involved in the discussions and actions as a group and are able to learn and grow. As the RDA’s mission is to build the social and technical bridges for data sharing and reuse across domains, has the COVID-19 WG itself been successful in breaking down walls and barriers, in combining strengths, and achieving a mutual goal? To capture participant perceptions of the extent to which engagement during the RDA COVID-19 WG activities was productive and sustainable, respondents were asked the following questions:

- B-2: Describe RDA COVID-19 WG as a collaborative forum (free form text input).
- C-1 and C-2: Rate your commitment and engagement level to the group/subgroup you belonged to (Likert-scale responses)
- C-3 and C-4: List the most challenging and rewarding aspects of the collaboration process (free form text input)

### 3.3 Survey and data collection

A draft version of the survey questionnaire was piloted by five people from the main working group and sub-groups to assess the intelligibility of the questions, especially in the section on radical collaboration, and to get general feedback about the survey regarding any ambiguity or bias. This led to some minor changes in the wording and presentation of the questionnaire.

The finalised questionnaire (see *Extended data*) was distributed via the RDA platform and a link was sent to the RDA COVID-19 WG and sub-WG’s by email via the WGs’ distribution lists. This included 164 members who had worked directly on the drafting of the recommendations and guidelines, 30 of whom are co-authors of the present paper. Nine of the 30 co-authors were also co-chairs or co-moderators of the RDA COVID-19 WG or sub-WG’s. There were no specific exclusion criteria.

### 3.4 Data analysis

Following the guidelines of ALLEA (2017), and as part of the RDA mission to “build the social and technical bridges” for collaboration, the analysis process focused particularly on respect for participants and mentoring. The co-authors of the present study included a variety of discipline specialists and a range of experience. The writing occurred in a shared Google ^TM^ document and the co-authors met at least weekly virtual meetings throughout all phases of the study. This provided opportunities for more experienced co-authors to share their experience and mentor others in participating and completing the research.

A mixed methods approach was adopted for data analysis. For quantitative analyses, the original responses were downloaded as a CSV file. Fields containing free-form text were extracted into an MS Word ^TM^ document for qualitative analysis. Extracts were tagged to identify common themes and concerns using the qualitative data analysis (QDA) computer software package NVivo ^TM^. Alternative software would include Taguette, a free open-source tagging tool.

The values for the Figures were generated from the anonymised dataset using OpenRefine (version 3.4.1 for Windows) and applying a text filter to the relevant variables. A first version of the Figures was then produced using the Insert/Chart function and the Chart Editor of GoogleSheets. These charts were then saved as SVG files and converted into EPS/TIF files using the free opensource software GIMP (version 2.10.22 for Windows).

***High level overview.*** A series of thematic maps were associated with the survey questions, providing a high-level overview of the types of issues identified when analysing the free-form text responses:

- RDA as a collaborative forum - positive experiences (Question B-2)
- RDA as a collaborative forum - negative experiences (Question B-2)
- How COVID-19 affected the ways of contributing to RDA (Question B-3)
- Summary of the sub-themes associated with *Challenges* (Question C-3)
- Summary of sub-themes associated with *Rewards* (Question C-4)
- Summary of sub-themes associated with *Ingroup* (Questions C-3, C-4)
- Summary of sub-themes associated with *Outgroup* (Questions C-3, C-4)

***Quantitative analysis.*** Questions A1-A10 were classificatory, and results provided socio-demographic characteristics of respondents (see Section 4.1). Frequency categories were reported as totals.

***Qualitative analysis.*** ‘Perceptions of inclusion’ relating to radical collaboration are reported in Section 4.2. Two experienced qualitative researchers analysed the free-form text responses. In the case of B2 to B4, an inductive thematic analysis approach was adopted to identify common themes based solely on what respondents wrote ( Braun & Clarke, 2006). The second set, C3 and C4, used an initial coding scheme with four codes: *Challenges* and *Rewards*, along with *Ingroup* and *Outgroup*, based on the overall theme of radical collaboration which informed the design of the survey. The codes *Ingroup* and *Outgroup* were chosen on the basis that feelings of inclusiveness and reduced defensiveness in a radical collaboration framework ( Tamm & Luyet, 2010) would be associated with the perception of *Ingroup* membership ( Hornsey, 2008). Such feelings may also promote identification of *Rewards*. Lack of inclusiveness, resulting in defensiveness, would lead to alienation and feelings of exclusion from the *Ingroup* (i.e. being part of an *Outgroup*). Identifying oneself as excluded from the *Ingroup* might be expected to result in an over-emphasis of the *Challenges* associated with the recent RDA collaboration.

Intercoder reliability was validated between the researchers as follows. Each researcher took responsibility for one of two groupings of responses:

- B-2 - B-4, which sought to explore participant perceptions of RDA collaboration; or
- C-3 and C-4, looking at more general perceptions of the collaborative experience.

The researchers then exchanged the outputs of their initial analyses to validate independently that they perceived the codings to be feasible. Codings were then cross validated through discussions with co-authors to modify any results on which researchers did not agree or to consolidate agreed findings.

The present work follows the SRQR guidelines for reporting qualitative research ( O’Brien *et al.*, 2014).

## 4. Results

Of the 69 respondents, 68 were retained for analysis. One respondent was excluded based on their free-form input to questions about their experience with the RDA COVID-19 WG collaborative effort. Instead of answering these questions, the respondent made very specific and highly critical comments about specific stakeholders related to the coronavirus pandemic, not the RDA WG.

The survey response rate was 11% of the RDA COVID-19 WG membership (68/600*100), or 41% of the subset of contributing authors (68/164*100). Both response rates are of the same order as other anonymous surveys ( Guo *et al.*, 2016; Nulty, 2008).

### 4.1 Socio-demographics

**Figure 2.**
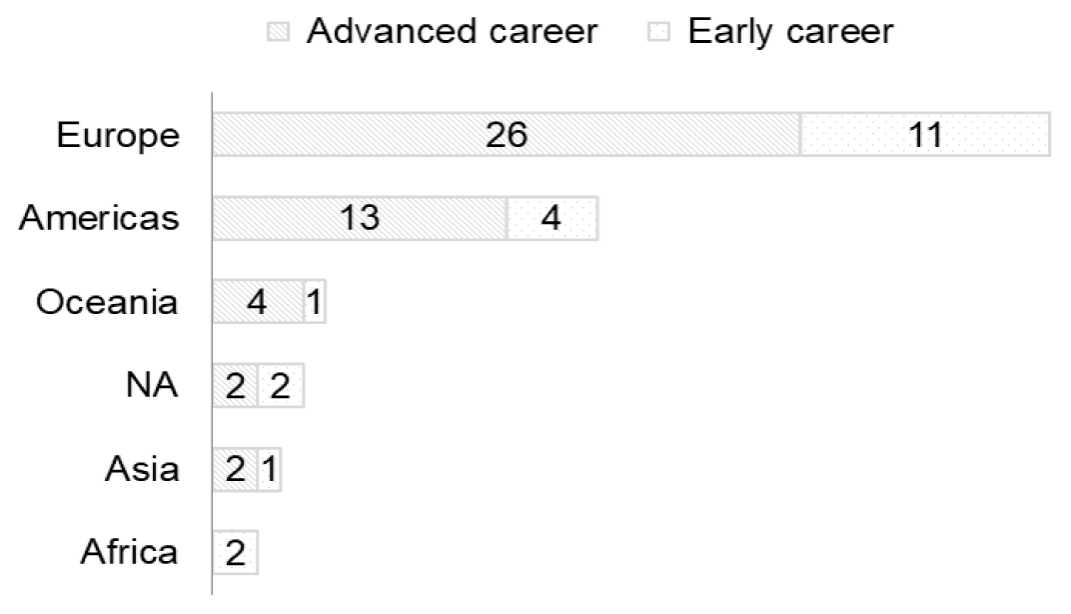
Region of residence of respondents. **NA** = missing (Question A-1+A-5).

The largest group of respondents was from Europe (n=37) followed by the Americas (n=17). Ten were spread around the world and four respondents did not indicate their region of residence ( Figure 2).

**Figure 3.**
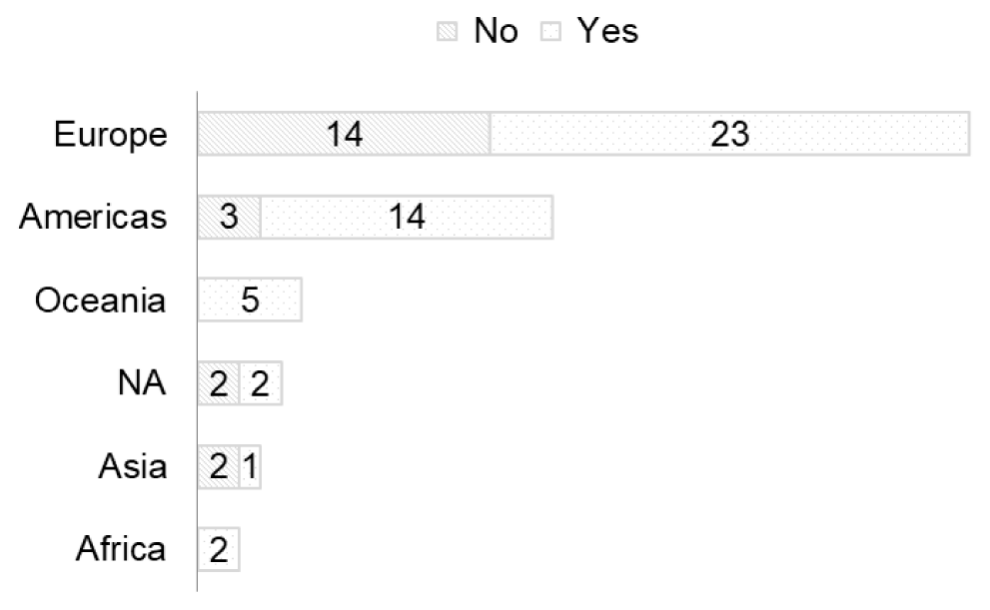
Region of residence of newcomers to RDA (Question A-1+A-6). Yes/No = were/were not members of RDA before joining the COVID-19.

The COVID 19 pandemic also brought a larger number of newcomers to the RDA. For example, 14/37 and 3/14 respondents from Europe and the Americas, respectively, became new members of RDA during the pandemic ( Figure 3).

**Figure 4.**
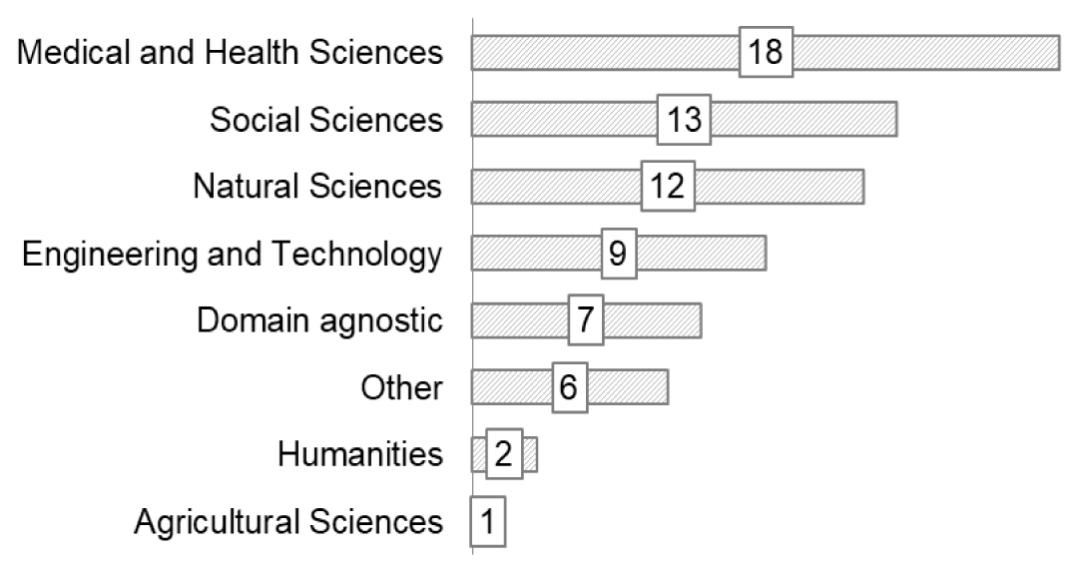
Domain of research/expertise of respondents (Question A-2).

The largest group of respondents were from the Medical and Health Sciences (n=18), followed by 13 from the Social Sciences ( Figure 4). Supporting areas such as engineering and technology, legal and ethical, community participation, and research software were less well represented.

**Figure 5.**
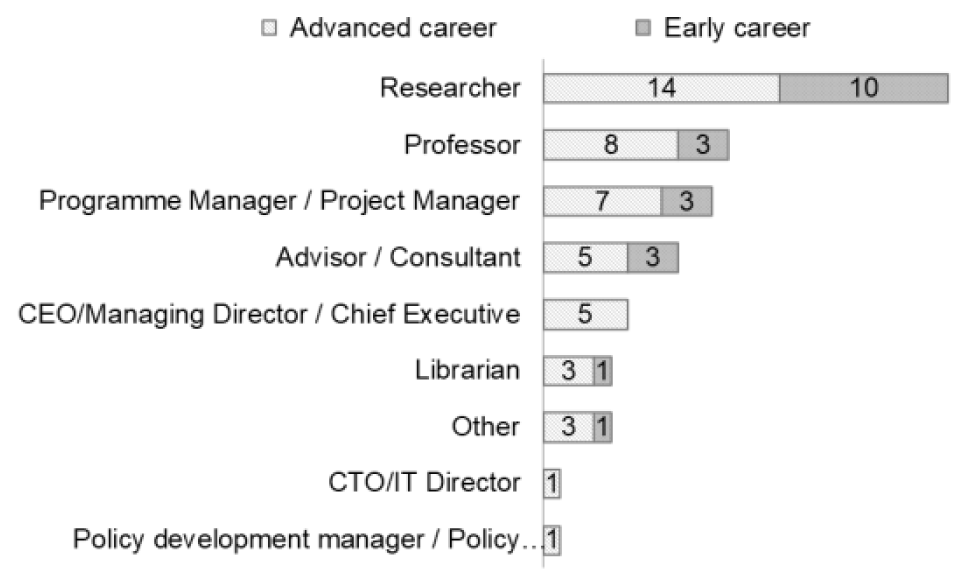
Professional role versus career stage of respondents (Questions A-3 & A-5).

**Figure 6.**
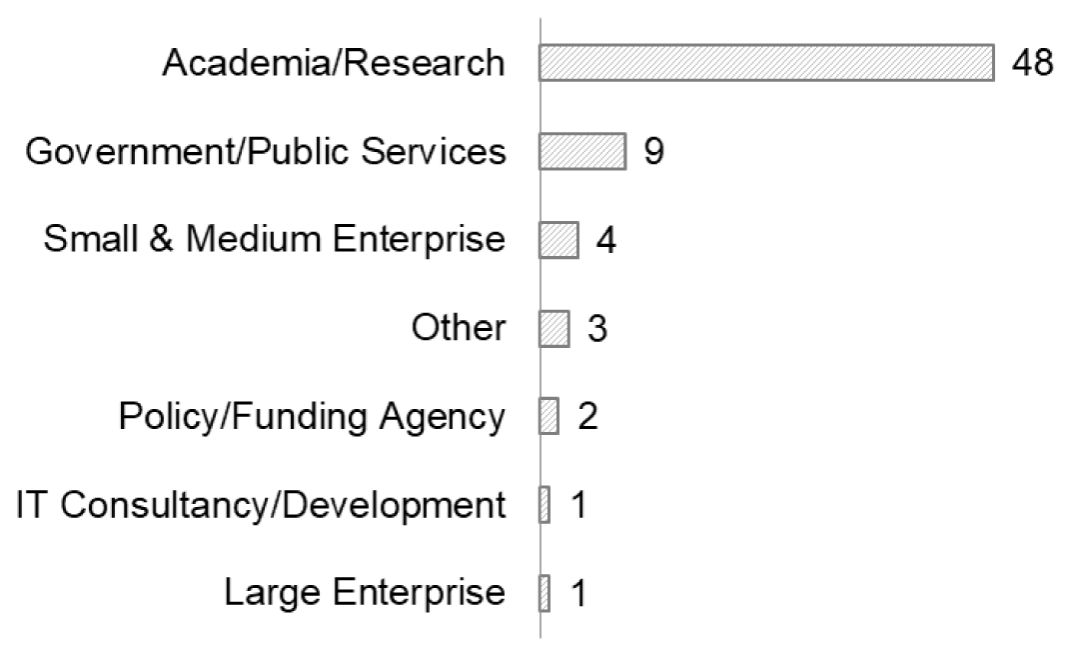
Organisation type of respondents (Question A-4).

Career types tended more towards academia, although some non-academic professionals took part. The career stage seems to have been evenly spread ( Figure 5). Respondents identified themselves primarily as researchers (n=24) or professors (n=11). Most respondents were domain experts: researchers (14 respondents at an advanced career stage, and 10 at an early stage) and professors (8 respondents at an advanced and 3 at an early stage) ( Figure 5), with most of them from academia/research organisations (n=48) ( Figure 6). Career stage was not an impediment to inclusiveness.

Although mostly from academic/research and government/public services sectors ( Figure 6), there were also some respondents from small & medium enterprises (n=4), large enterprises (n=1), and policy/funding agencies (n=2). Their presence is a good indicator of inclusiveness (see Section 4.2 Perceptions of inclusion) and an interest in open data sharing beyond the research context, as evidenced elsewhere by the Open COVID Pledge. It suggests that the RDA can involve significant actors from all related sectors (funding, research, government, business) ( Figure 6).

**Figure 7.**
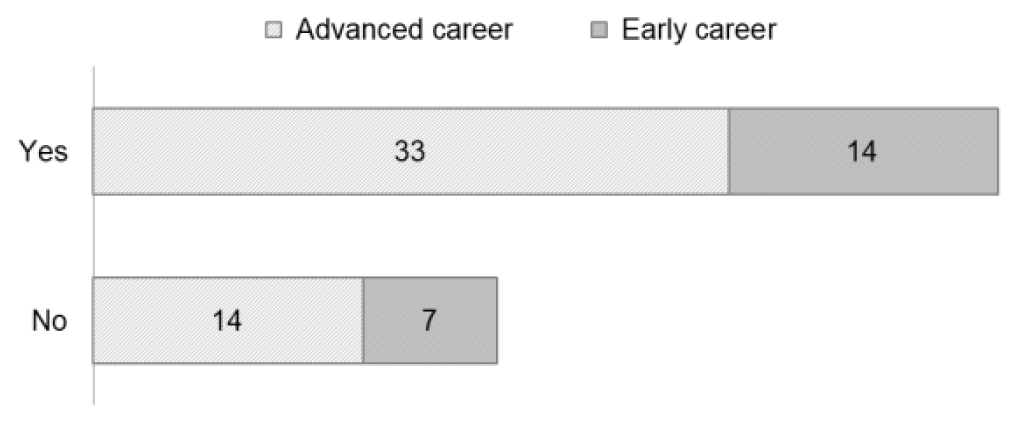
Career stage of respondents in relation to prior involvement in RDA (Questions A-5 & A-6).

Forty-seven respondents were a member of the RDA before joining the COVID-19 WG (the *Yes* line in Figure 7). Most advanced career persons were also already members of the RDA community (33 persons). Twenty-one persons at various stages of their career became new RDA members through their involvement in the COVID 19-WG.

**Figure 8.**
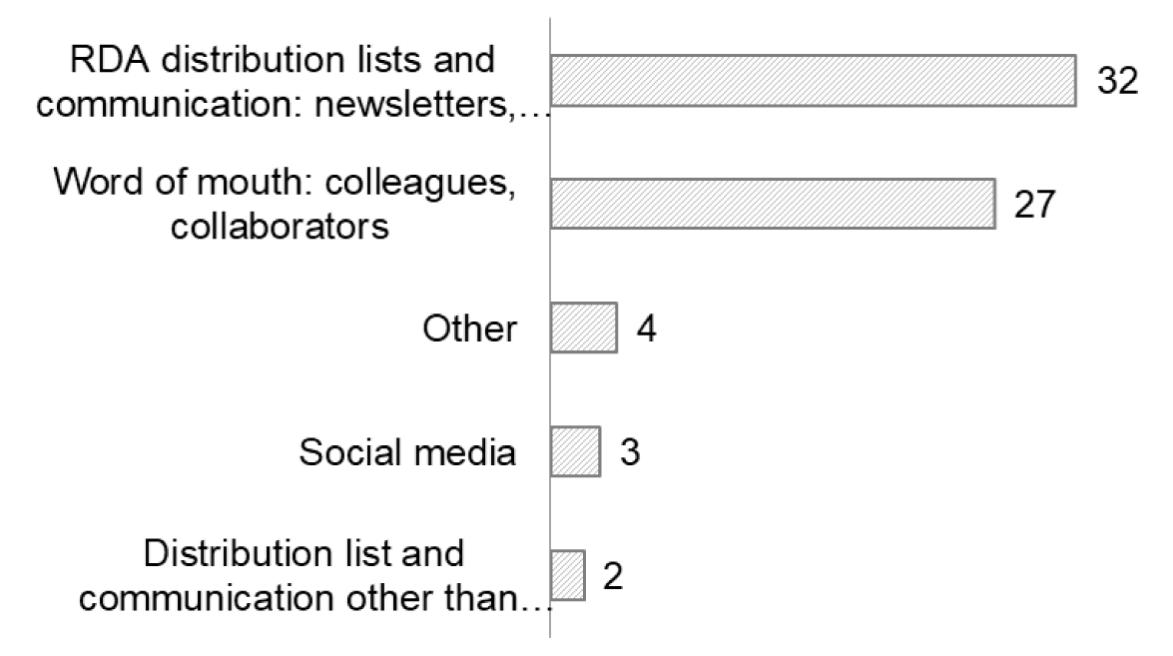
How participants learned of the RDA COVID-19 WG (Question A-8).

Thirty-two participants learned of the RDA COVID-19 WG via “RDA distribution lists and communications: newsletters, news items”. The second most frequent medium (27 respondents) was “Word of mouth: colleagues, collaborators” ( Figure 8). No striking differences were observed between early career and advanced career researchers with respect to this question ( Figure 8).

**Figure 9.**
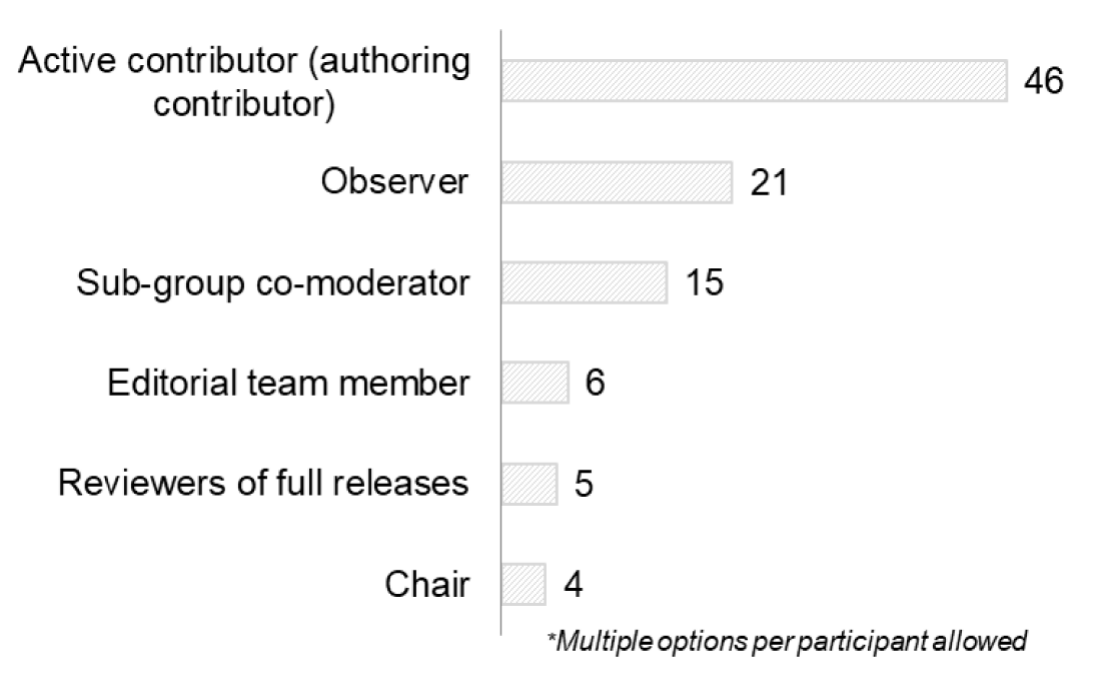
Role of respondents in the RDA COVID-19 WG (Question A-9).

Most respondents were active contributors (n=46) followed by observers (n=21) and sub-group co-moderators or chairs (n=19) ( Figure 9). Respondents could select more than one option.

**Figure 10.**
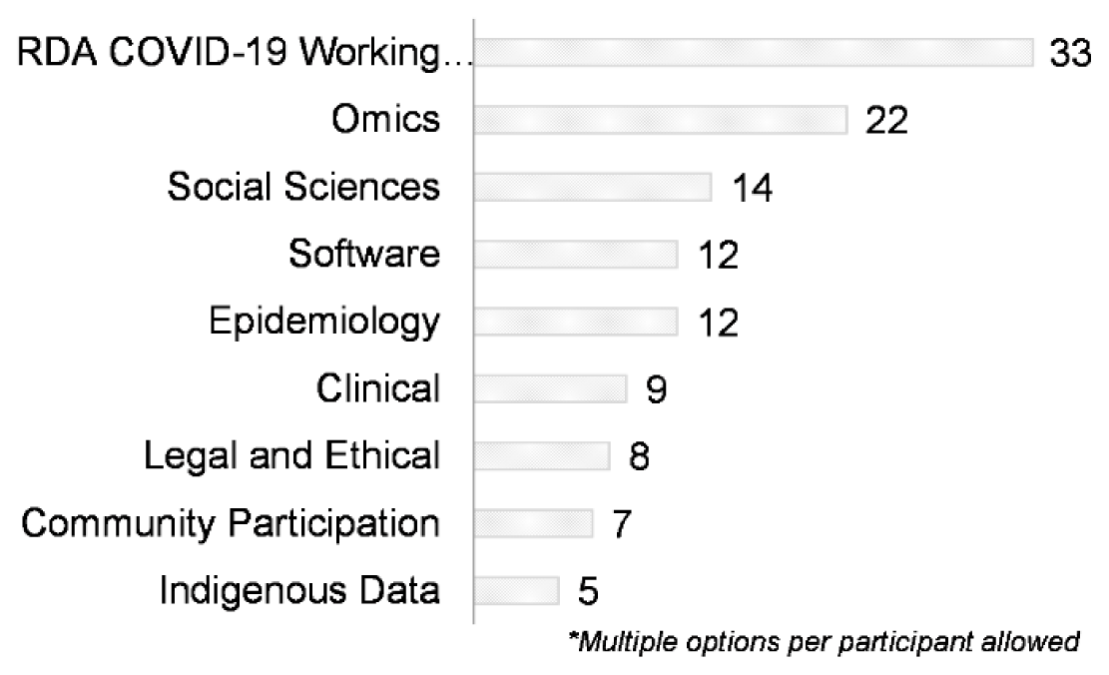
Participation in the RDA COVID-19 WG and sub-groups (Question A-10).

Thirty-three respondents were members of the overarching RDA COVID-19 WG followed by the sub-WGs Omics (n=22), Social sciences (n=14), Epidemiology (n=12) and Software (n=12) ( Figure 10). Note that respondents could select membership in the RDA COVID-19 WG alone and no sub-WGs, or one or more sub-WGs. 

### 4.2 Perceptions of inclusion

**Figure 11.**
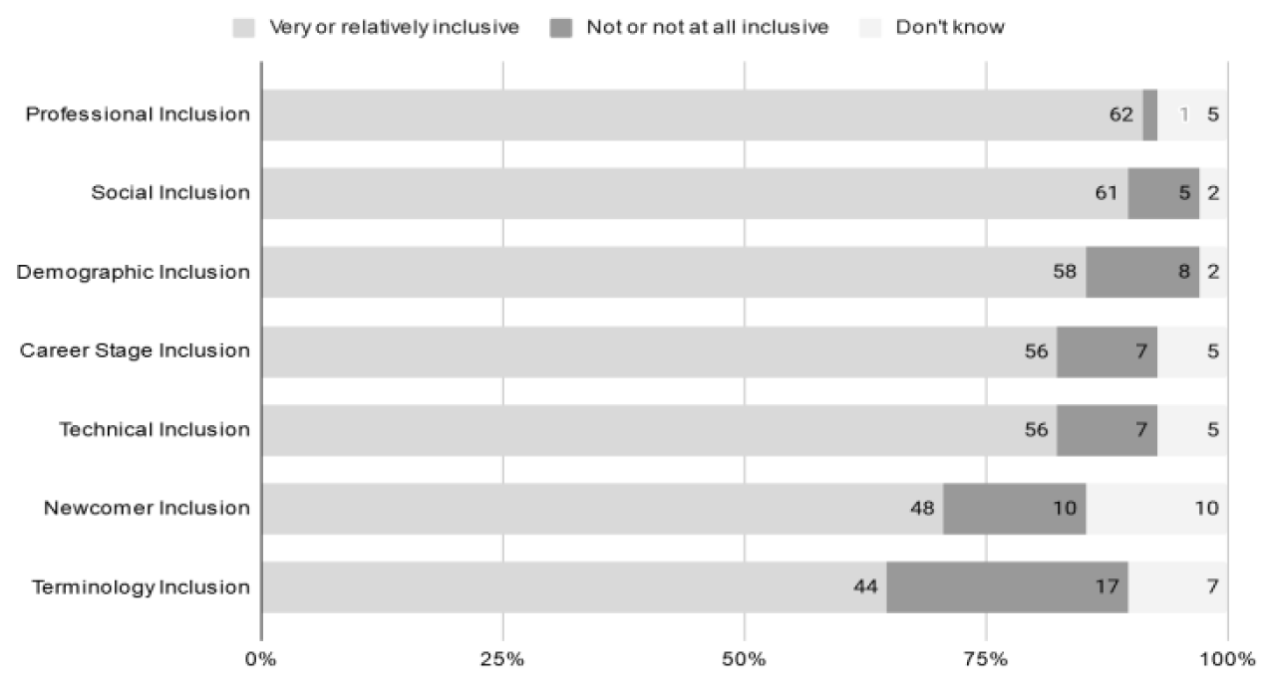
Cumulative scores relating to inclusion across different dimensions. Numbers represent those reporting Very inclusive or Relatively inclusive (out of 68 total responses) (Question A-11).

Responses to question A-11 pertain to perceived levels of inclusion in the collaborative effort as described by McGovern (2018a, p14). Most survey respondents reported positive inclusion (i.e., *very inclusive* or *relatively inclusive*) across all dimensions – social inclusion, demographic, professional, career stage, RDA membership (newcomer), technical platform (connection to the virtual meetings) and terminology (the language used during discussions and in the resulting guidelines) ( Figure 11). 

**Figure 12.**
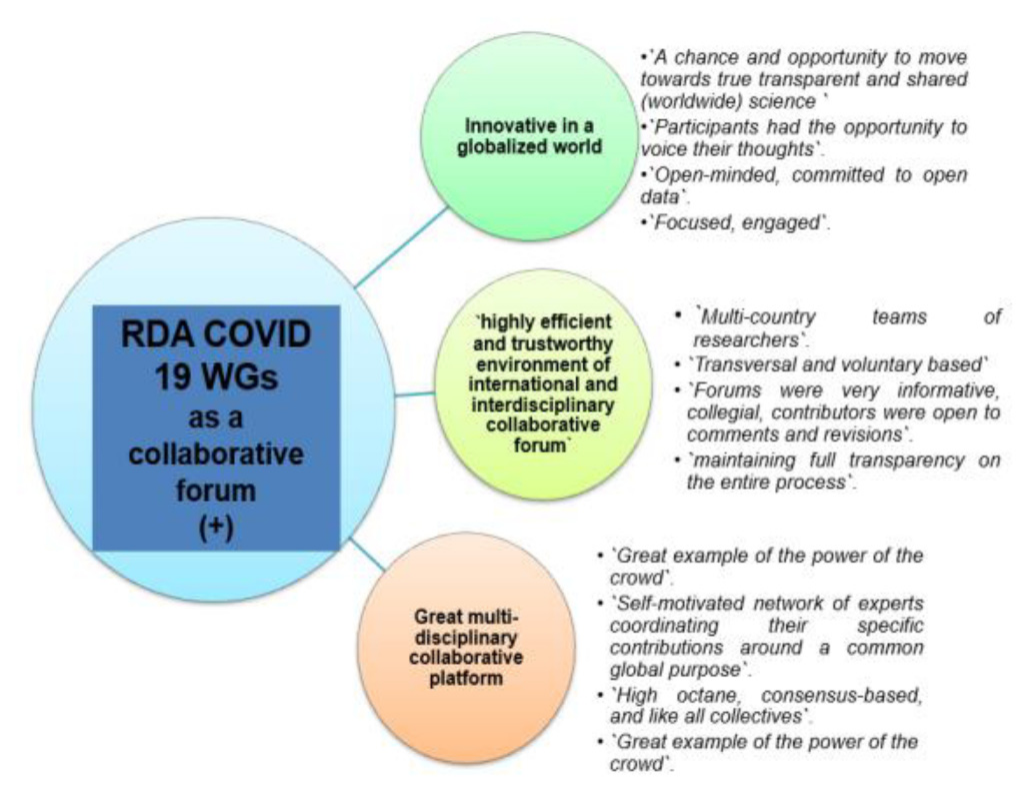
Positive (+) experiences of the RDA as a collaborative forum (Question B-2). Examples of free text responses shown as bullets.

**Figure 13.**
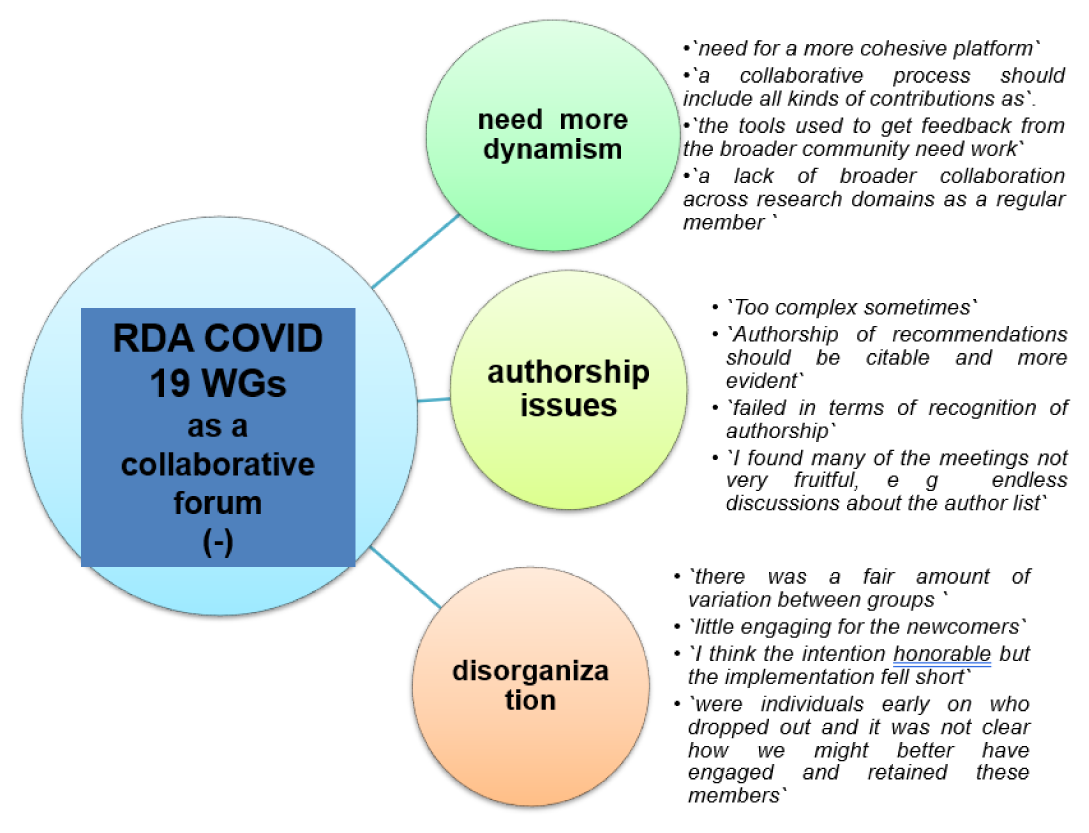
Negative (-) experiences of the RDA as a collaborative forum (Question B-2). Examples of free text responses shown as bullets.

Respondents used positive (+) or negative (-) adjectives to describe the RDA COVID-19 WG as a collaborative forum (Question B-2). Only 3/38 respondents skipped this question. There emerged three positive themes (“ *innovative in a globalised world*”, “ *highly efficient and trustworthy environment of international and interdisciplinary collaborative foru* *m*”, and “ *great multidisciplinary collaborative platform”*), and three negative (-) themes (“ *need for more dynamism”, “authorship issues”* and *“disorganization”*) ( Figure 12 and Figure 13).

Analysis of responses to B2-B4, and C3 and C4 emphasises the categories *Professional, Social, Demographic* and *Career-Stage Inclusion* presented in Question A-11. Respondents highlighted a sense of belonging in terms of their contributions to overall goals, being heard, and having access to expertise in other disciplines that they would not normally have expected. Multiple relevant sub-themes were identified associated with the initial codes, *Rewards* and *Ingroup*. For *Rewards*, saturation of sub-themes was reached after analysing four of the 68 responses. For *Ingroup*, saturation was reached with seven out of 68 respondents ( Fusch & Ness, 2015). This suggests good overall agreement across respondents and that the sub-themes identified salient issues for them ( Figure 12 and Figure 13).

Respondents reported positive perceptions of a sense of community and working together:

“To see that there is a wide group of people that it does [ *sic.*] their bigger efforts to listening all the voices and consider that everybody has something interesting and important to say” (CP15789 ^1^)

“Participants had the opportunity to voice their thoughts” (BP15921)

In addition to recognition of the power of collaboration, respondents explicitly signalled their own sense of *Ingroup* identity, using pronouns such as “we” and “us”:

“We succeeded!” (CP15881)

validating the sense of a common achievement. They recognised that working together in this manner was important in ensuring successful outcomes:

“It needs more traction among people like us” (CP15923)

Beyond the achievements of the RDA initiative, participant feelings of belonging extend to the level of personal interaction:

“... being connected and having the impression of "old friends" with many people I have never met outside virtual meetings!” (CP15329)

“Forums were very informative, collegial, contributors were open to comments and revisions” (BP15957)

an experience which offered new opportunities:

“[we] got to interact with people I would never meet otherwise” (CP15870)

and opened up new horizons:

“… and discover new worlds” (CP15870)

via a cross-disciplinary environment which might otherwise not have been available:

“On one day I had a call with Software Engineers and another with people from a Law background I treasure that” (CP15928)

They could even become defensive regarding external or internal threats. For instance, *Ingroup* activities may be contrasted favourably against what other groups ( *Outgroups*) might be doing or thinking:

“Another challenge was our group adhered closely to the format provided (and timeline) but other groups did not as much” (CP15927)

or there could be a need to be protective of the group against internal dissent:

“Also, certain individual members kept pushing arguments that were clearly already decided by the larger group, and this added unnecessary time wasting” (CP15939)

Inclusiveness was not only evident in terms of *Ingroup* membership, informal or otherwise. It also became apparent when participants referred to the outcome. There was a sense of pride, for instance, that people worked toward a common goal:

“Being able to provide concrete and actionable recommendations that the research community needed” (CP15926)

even a tribute to inclusive collaboration:

“Great example of the power of the crowd” (BP15954)

There was ultimately an emotional drive associated with RDA collaboration during the unfolding pandemic:

“It is a service to God and humanity” (CP15971)

These perceptions, as expressed in response to Questions C3 and C4, echo more explicit themes in questions B2–B4. There was a level of coherence, therefore, in how participants felt about contributing and being part of the RDA COVID-19 WG effort.

**Figure 14.**
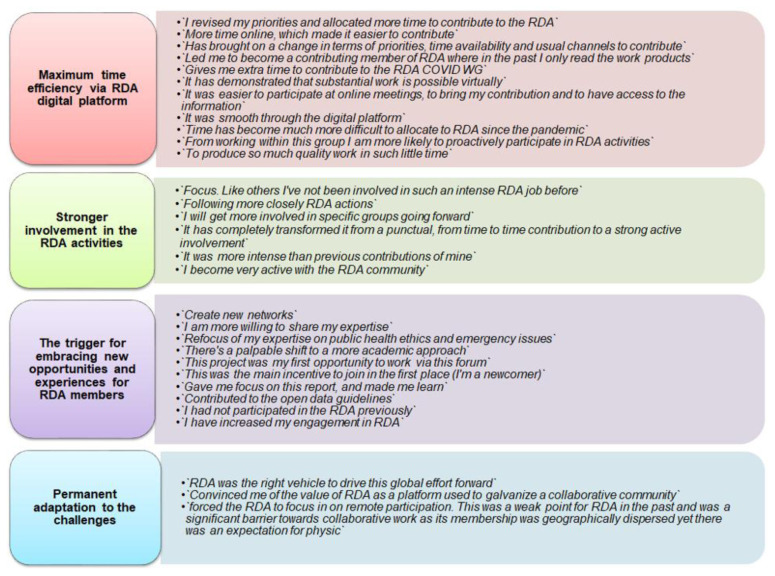
B-3. How COVID-19 affected the ways of contributing to RDA (B-3).

Regarding the influence of COVID-19 on the manner of contributing to the RDA, respondents focused mainly on the fact that during the pandemic they had a `stronger involvement in the RDA activities` because they needed to keep contact with counterparts from different countries. Online meetings saved on travel time and may have contributed to greater commitment. There were an important number of respondents who considered that the pandemic acted like the trigger for embracing new opportunities and experiences for RDA members. They found opportunities to create new networks and establish new connections with specialists from different disciplines from around the world that they would never have had the opportunity to meet otherwise ( Figure 14). Several respondents also maintained that the pandemic had no influence on their previous involvement or activities and they still kept their prior work style.

For the RDA COVID-19 initiative, stressing the overall vision and encouraging support and engagement with that vision were clearly important. The pandemic provided an external stimulus for experts to engage and ‘do their bit’ when otherwise unable to take control back and manage their own lives. This would be an extrinsic motivator and therefore remain short-lived. Elsewhere, however, respondents reported longer lasting outcomes more indicative of intrinsic, enduring changes and motivations:

“[I will now] refocus ... my expertise on public health ethics and emergency issues” (BP15963)

and encouraging early-career researchers to engage:

“This project was my first opportunity to work via this forum” (BP15921)

In such cases, RDA’s facilitation of inclusive collaboration validated a sense of purpose despite other contextual challenges in a post-truth era:

“it has brought a lot of benefit personally - in opening up to different ways of working and reinforcing the perception of the 'good' in the research community, especially during times when not only politicians but also 'experts' in all fields are under suspicion I would be very happy to continue and extend such collaboration in future” (BP16030)

Inclusiveness was, therefore, an essential feature of radical collaboration and can be evaluated across multiple dimensions.

### 4.3 Distributed digital practices

As McGovern (2018a, p.16-18) observed, collaboration has increasingly become long-distance and distributed. During the pandemic, this was imposed. Nevertheless, the digitalisation of many work environments was already in place. Without it, the RDA COVID-19 WG initiative would not have been able to engage so many experts worldwide. Along with that came the challenges McGovern (2018a, p.16-18) described in supporting a diverse community with differing levels of technical competence and experience with this way of working. What she did not consider during pre-pandemic writing was the sense of isolation and aloneness imposed by remote working and social distancing ( Jetten *et al.*, 2020). The RDA COVID-19 WG initiative was also nominally organised along agile development lines with week-long writing sprints and weekly virtual coordination meetings. The survey sought, therefore, to explore multiple aspects of how respondents perceived WG and sub-WG organisation and processes.

**Figure 15.**
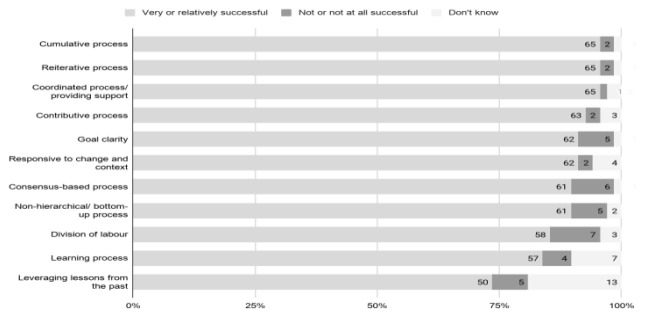
The perceived success of distributed digital practices (Question B-1).

Question B-1 asked participants to rate how successful the working group was in implementing and supporting distributed digital practices with respect to cumulative and reiterative processes, responsiveness to change and context, learning process, leveraging lessons from the past, contributive, consensus-based, non-hierarchical/bottom-up, and coordinated process as well as goal clarity, and division of labour. The results are shown in Figure 15 where the four-point Likert scale options are reported as totals for the positive perceptions (‘Very successful’ and ‘Relatively successful’) and negative perceptions (‘Not successful’ and ‘Not successful at all’), with the ‘Don’t know’ option in addition.

Figure 15 illustrates the overall perception among the 68 respondents of the success of distributed digital practices. Across all eleven dimensions, the vast majority (with scores ranging from 57 to 65) indicated the distributed digital practices were *Very successful* or *Relatively successful,* with the highest scores (65 each) obtained for *Cumulative process*, *Reiterative process* and *Coordinated process/providing support*. An only slightly lower level of success (50 positive responses) was reported for *Leveraging lessons from the past.* Considering the relatively large number of *Don’t know* responses (13 *Don’t know*, as opposed to 5 negative responses), one partial explanation for this finding might be the nature of the effort in terms of the unprecedented urgency, short time span and scale.

**Figure 16.**
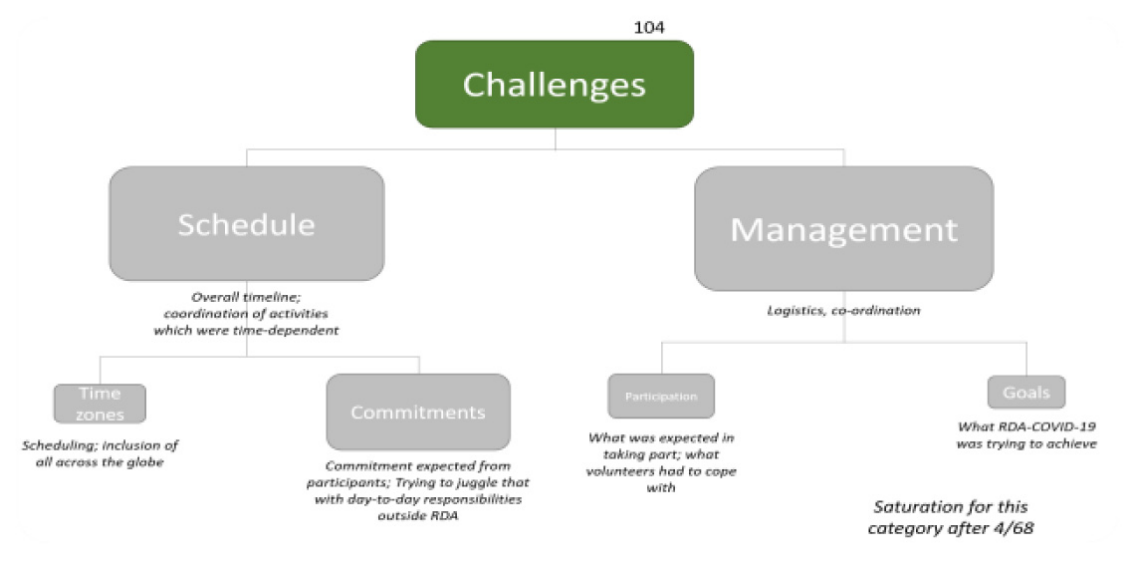
Summary of the sub-themes associated with Challenges (C-3).

In their free-text responses, respondents recognised specific challenges associated with organising this type of collaboration and reported more personal issues ( Figure 16). At the same time, they recognised the supportive and encouraging aspects of the RDA framework to facilitate the time-critical, global collaboration. Some respondents highlighted scheduling problems due to time zone differences which limited participation and on occasion led to feelings of exclusion:

“Time zone differences - I was able to stay up late to attend some of the meetings, but the work was carried out by others when I was asleep” (CP15969)

Beyond issues of participation, however, respondents highlighted that the tight deadlines imposed significant challenges.

“The tight timeline was also challenging” (CP15926)

and

“The speed with which the work was undertaken with weekly deliverables” (CP16044)

With a slight hint of defensiveness, some felt this led to compromises in quality

“[...] had there been more time, I have no doubt that the overall quality of the final output would have been even higher” (CP15926)
  “Not enough time to keep up with all the literature that was also rapidly developing, creating opportunities for errors and omissions” (CP16044)
  “the work was carried out by others when I was asleep, meaning I couldn't discuss things with anyone on-the-fly” (CP15969)

and restricted participation:

“Being present and involved as much as this initiative required” (CP16021)
  “Keeping up with the many calls, deadlines, documents, rolling notes” (CP15932)
  “And it is very hard for everyone to participate if a meeting is held only once” (CP15930)

There were also perceptions that some approaches to coordination restricted opportunities for all to participate to the level they might have hoped:

“Frustration of many who were not used (and could not adapt) to the seemingly chaotic way of producing a text” (CP15870)
  “There were individuals early on who dropped out and it was not clear how we might better have engaged and retained these members” (BP15927)

With respect to the challenges associated with the circumstances of distributed collaborative practices, some technical barriers were identified.

“I have many cases of internet connection problem” (CP15924)
  “Unstable internet connection can be a hindrance Sharing files online or via Google Drive can address this gap” (CP15971)

Taking scheduling, overall management, time zones and so forth together, one participant summed it all up as:

“zoom fatigue” (CP15921)

echoed elsewhere:

“I think the intention honourable, but the implementation fell short” (BP16055)

Comments like these represented the many aspects which must inevitably be addressed with such an initiative. However, over time collaborators can adapt. Whatever the constraints with distributed processes, respondents recognised that the collaboration experience was positive:

“[...] the massive involvement of most participants and the always - even under clear stress - friendly tone and open atmosphere” (CP15932)

And, ultimately, innovative and productive:

“The highly interesting exchanges with the other group members and how consensus was obtain [ *sic.*] and also how new ideas were developed” (CP15958)

There was a willingness to make do and look for the benefits of distributed collaboration, which participants were quick to recognise:

“I had lots of fun because I learnt a lot and got to interact with people I would never meet otherwise, and discover new worlds” (CP159870)
  “I also had the opportunity to develop an understanding of disciplines very different from my own” (CP15978)

Indeed, whatever the challenges for distributed collaboration, this sort of initiative bodes well for open science and future collaborative efforts:

“I believe that this experience, developed out of an urgent need and call for action, will help us to collectively get over that discomfort and learn how to embrace open data and open science” (CP16044)

Participants were very aware of the challenges of remote, distributed collaboration as described by McGovern (2018a, p.16–18). However, there were also indications that respondents adapted and were willing to overcome those challenges given the possible gains. Exploration of the dimension of inclusiveness concluded that any negative aspects and potential *Outgroup* perceptions were moderated by the opportunities and rewards afforded by collaboration. McGovern (2018a, p.29) stressed that collaborators may need to adapt, but without considering the mechanisms which motivate such adaptation. The results of the present study with respect to distributed digital practices echo the results for perceptions of inclusiveness – a willingness to engage and accept the inconveniences and challenges of remote, distributed collaboration when offset by potential gains: (a) the reach of a virtual environment in terms of who can engage and therefore who comes into contact with whom; and, (b) the opportunity to learn and play a role in addressing an emergency situation – all the while showing higher tolerance and availability.

### 4.4 Collaborative engagement and commitment

**Figure 17.**
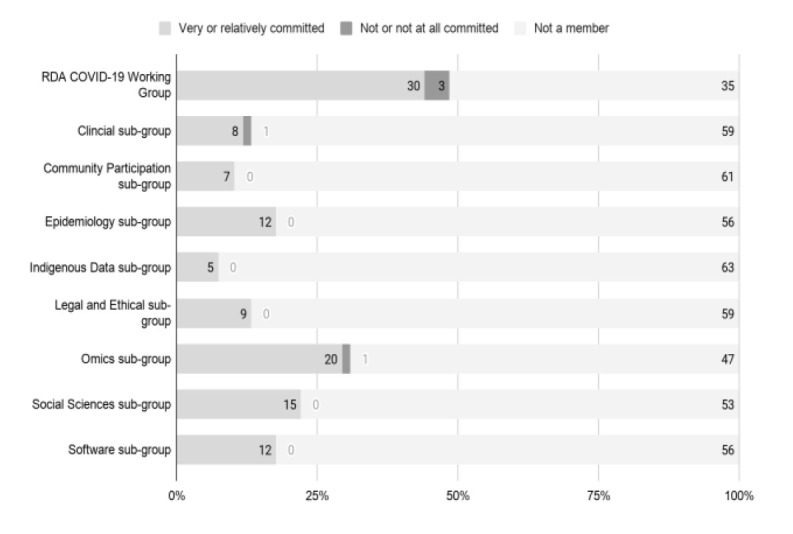
Communal commitment of the RDA COVID-19 WG and subgroups. (Question C-1).

**Figure 18.**
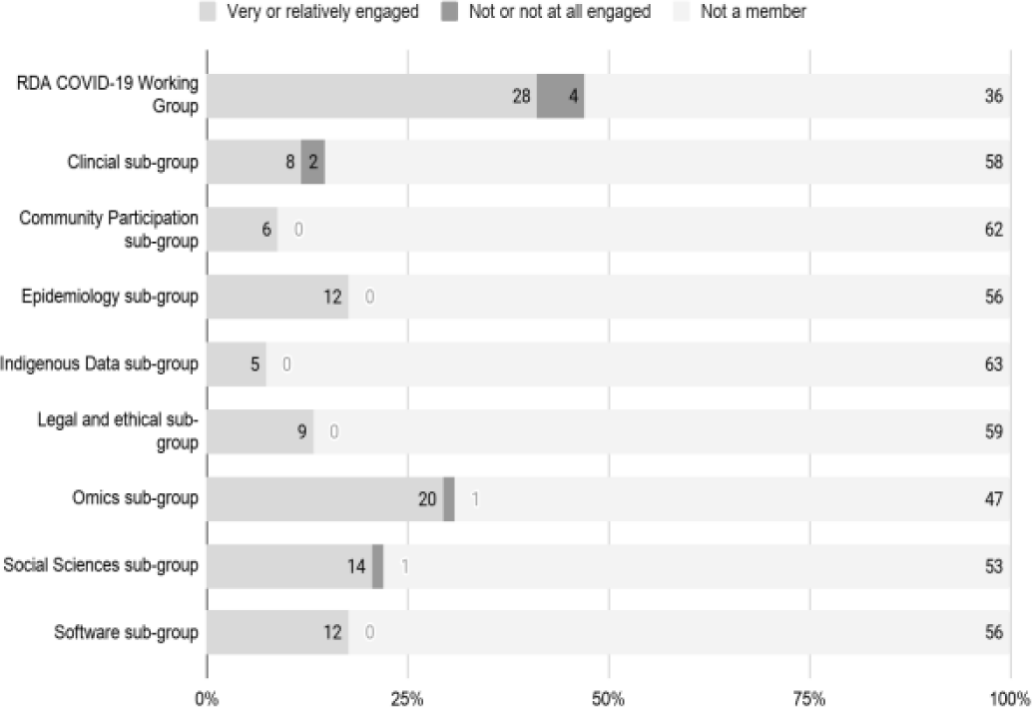
Interactive engagement of the RDA COVID-19 WG and subgroups (Question C-2).

The positive response to the RDA COVID-19 WG collaborative experience informed the third main construct in McGovern’s account of radical collaboration. In addition to commitment ( Figure 17) and engagement ( Figure 18), respondents reported their experience of the RDA environment (B2-B3) and of the collaboration (C3, C4) as free-form text in response to open-ended questions. As described below, many observed that the networking opportunities provided by the RDA had led to other benefits beyond the specific RDA recommendations and guidelines. With only a few exceptions, and regardless of sub-working group, respondents almost exclusively reported high levels of commitment ( *Very committed/Relatively committed*) ( Figure 17) and engagement ( Figure 18).

Respondents were self-selecting and so are likely to have been more motivated to engage. Nevertheless, considering that this international and interdisciplinary collaboration took place over an intensive period with frequent meetings, while many were dealing with changing work and family-related structures and processes, illness (including self, family members, or close friends falling ill with or recovering or dying from COVID-19), lockdown situations including home-schooling, summer holidays, and so forth – it is quite remarkable that many still perceived the commitment to be very high or relatively high.

**Figure 19.**
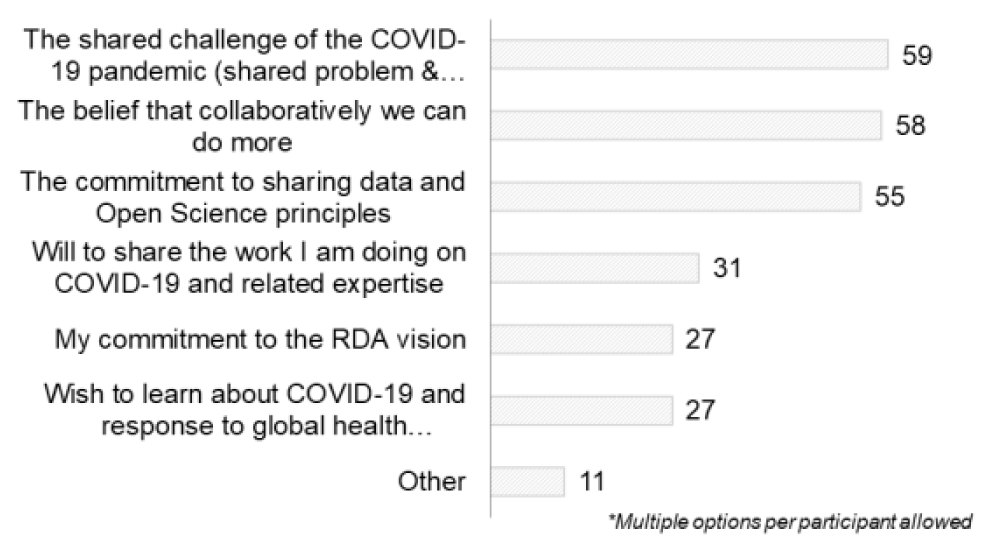
Motivation for getting involved in the RDA COVID-19 WG (Question A-7).

Question A-7 asked respondents to select all the relevant factors that brought them to the COVID-19 WG. The main motivation for becoming involved was the shared challenge of the COVID-19 pandemic (shared problem/common goal) (n=59) and the belief that collaboratively we can do more (n=58), followed by the commitment to sharing data and open science principles (n=55) ( Figure 19).

The external factors – namely the pressures introduced by the pandemic – represent extrinsic motivators ( Deci & Ryan, 2000), and so were short-lived, declining once the external context changed. In the present study, respondents were also internally motivated. They had engaged because they felt that collaboration and sharing data were appropriate things to do. To some degree, this view was consistent with other comments such as concern about recognition of authorship, although the latter does not seem to have affected the level of engagement.

**Figure 20.**
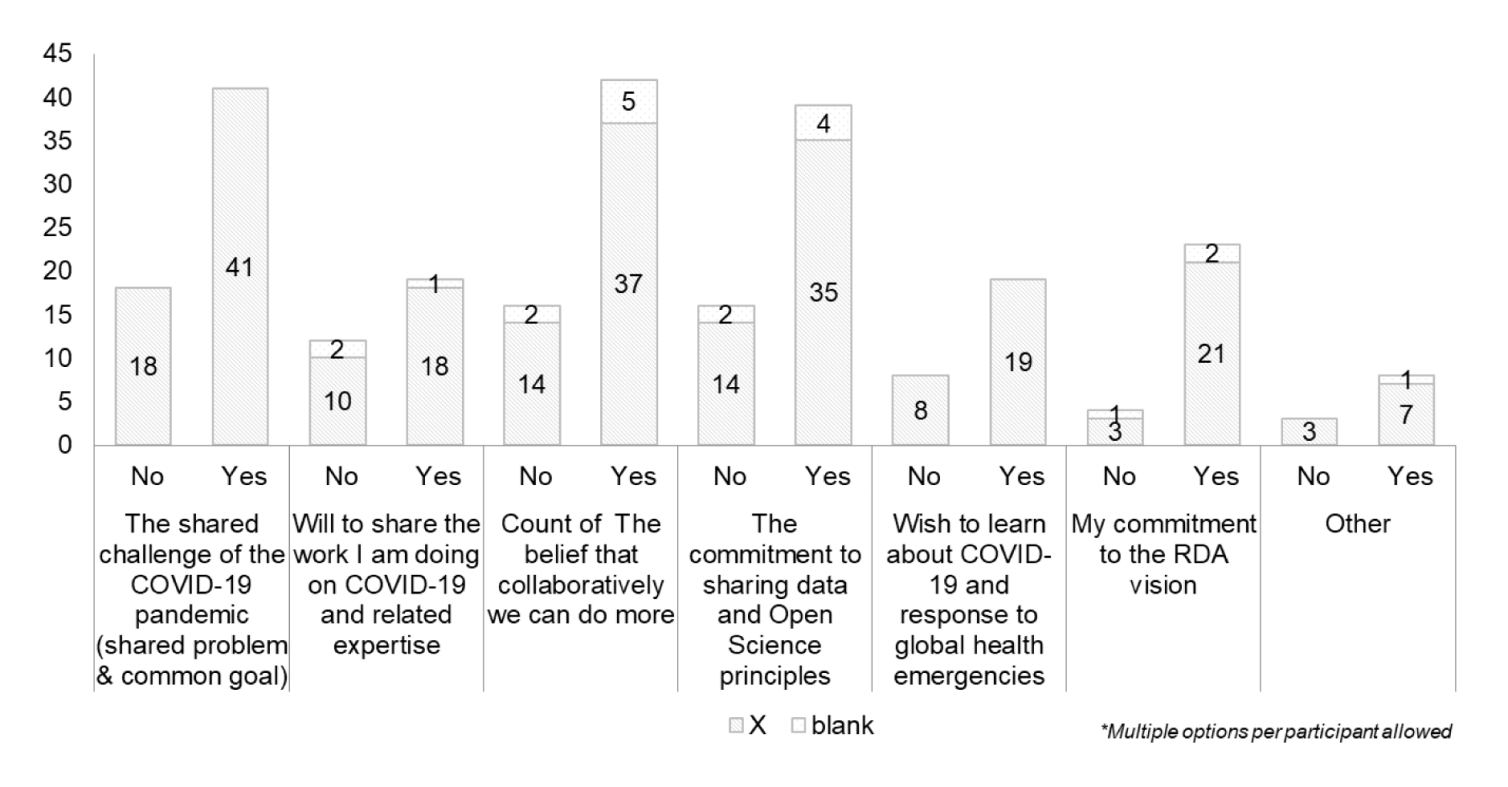
Relation between motivation of getting involved in RDA for newcomers or members of RDA (Question A-6+A-7).

For both previously engaged RDA members and newcomers, “ *common goals*”, “ *belief that collaboratively we can do more,*” and “ *commitment to the RDA vision*” were the top three ranked options ( Figure 19 and Figure 20).

Commitment levels were also reflected in the free-form text input in B-2 - B-4, and C-3 and C-4, where respondents provided greater detail on their perceptions of the RDA COVID-19 WG and sub-working groups as a positive, inclusive collaborative experience. Comments covered a range of aspects relating to the opportunities afforded by the RDA COVID-19 WG collaboration, such as networking, acquiring new skills, and the opportunity to work with experts in their field with whom they may not otherwise have met. They described working together on the recommendations and guidelines very much as an opportunity:

“[to] discover new worlds” (CP15870) by “meeting experts, networking, learning” (CP16041)

These positive indicators suggest mitigation of negative aspects reported in responses to B2. For instance, there were specific challenges with respect to organisation:

“[There was] little engaging for the newcomers” (BP16058) and there needs to be some attention to the collaborative environment: “I think the intention honourable but the implementation fell short” (BP16055)

as well as concerns about recognition of authorship:

“Authorship of recommendations should be citable and more evident” (BP15961) and ultimately there were concerns it “failed in terms of recognition of authorship” (BP15962) which became disruptive on occasion: “I found many of the meetings not very fruitful, e.g. endless discussions about the author list” (BP16050)

Nevertheless, any such concerns did not detract from other kinds of opportunity but rather enhanced previous experience:

“I read about other countries' RDM research and development, but it feels very different when experts from those countries are presenting them, they add to the works and resources a personal and lively [angle]” (CP15959)

Beyond the networking opportunities afforded by the collaborative activities, it was felt that this was an opportunity to practice open science:

“this experience, developed out of an urgent need and call for action, will help us to collectively get over that discomfort and learn how to embrace open data and open science” (CP16044)

inspiring confidence for any future work they may do:

“For subsequent collective writings I have led, when people say it won’t work, I point to RDA COVID-19” (CP15870)

whilst recognising that something special was happening:

“To see that there is a lot of people that is [ *sic.*] happy to devote part of their productive time to this kind of initiatives (even without receiving any payment for it)” (CP15879)

and being part of something significant:

“The feeling that perhaps one is making a difference on something very important” (CP15928)

but at the same time, familiar and comforting:

“being connected and having the impression of "old friends" with many people I have never met outside virtual meetings!” (CP15925)

These feelings of kinship even provided cathartic or restorative benefit during the uncertainty of the pandemic itself:

“having a way to contribute to solving a global problem, even if indirectly, gave us a sense of control and meaning during very challenging times” (CP15934)

and a sense that we are not alone and that everyone has a contribution to make:

“To see that there is a wide group of people that it [ *sic.*] does their bigger efforts to listening all the voices and consider that everybody has something interesting and important to say” (CP15879)

There is little doubt that the challenges imposed by the short timelines, collaboration across different disciplines and with people at different career stages and levels of expertise might well have failed. However, not only was it the case that contributors quickly felt part of a significant collaborative effort, but they experienced additional benefits, not least of which was the protective support of *Ingroup* membership.

### 4.5 Additional findings

**Figure 21.**
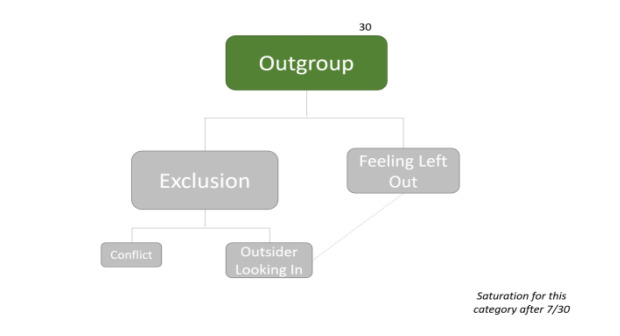
Summary of sub-themes associated with Outgroup.

As previously outlined, the online survey was structured around the three main constructs of McGovern’s (2018a) description of radical collaboration. Both the quantitative and qualitative analyses found support as outlined in the three previous subsections. However, the qualitative analysis revealed additional factors of the collaboration described below. Additional factors helped explain the psychological mechanism of a high level of commitment and engagement despite working with a diverse group of people and in a distributed digital environment. Four superordinate codes were used to analyse the free-form text responses for C3 and C4: *Rewards* and *Challenges* taken from the wording of the questions themselves, and *Ingroup* and *Outgroup*, motivated with respect to defensiveness. The rationale for this was that feeling part of a group ( *Ingroup*) would encourage reduced defensiveness, whilst alienation from the group ( *Outgroup*) might have had the opposite effect ( Figure 21). The nature of the motivation was briefly touched on in the previous section in relation to commitment and engagement ( Figure 17 and Figure 18).

Participants did highlight *Outgroup* feelings, especially with respect to joining or scheduling:

“Its [ *sic.*] hard to join a community already in flight and not feel like you're going over old ground, and frustrating the process, due to a lack of tacit knowledge” (CP15970)

With concerns that some of the discussion was monopolised by individuals and tended to focus on trivialities:

“I'm questioning my level of commitment because I perceive neither I nor others are heard or respected under certain circumstances” (CP16030)

Finally, there was also a concern that lack of expertise would be an inhibitor:

“I did not dare to actively collaborate because I felt I was not enough of an expert” (CP15973)
  “Figuring out what to say that was novel and not simply re-stating standard RDM guidance” (CP15976)

More general challenges around organisation and authorship had already been raised in response to B2 and as described above, but participants were clearly willing to compromise.

“It has been an immensely rewarding experience to be part of such a rapid coming together of experts contributing and learning from each other to achieve a common goal” (CP16044)
  “Contributing to such an important document in such a timely manner” (CP15922)

The significance of the aims of the working groups not only encouraged compromise regarding negative aspects of the experience, but led to a feeling of belonging ( *Ingroup* membership):

“Seeing the commitment of people to a common goal” (CP16051)
  “Social inclusion (irrespective of anything)” (CP16053)

Consistent with perceptions of inclusiveness described above, there was a sense of pride in what was achieved:

“The feeling that perhaps one is making a difference on something very important” (CP15928)
  “a feeling I might be able to help” (CP16055) and that “[...] we've made a difference” (CP16030)

and summing up:

“This was meaningful work” (CP16044)

Participants also recognised that taking action encourages self-efficacy:

“feeling to have contributed at least a little to coping with the pandemic” (CP16052)

which might even provide some level of solace amidst the confusion imposed by the pandemic:

“Also, having a way to contribute to solving a global problem, even if indirectly, gave us a sense of control and meaning during very challenging times” (CP15934)

Some individuals found it difficult to feel part of the working group, either because they felt they had nothing to contribute or in response to difficult – what Tamm & Luyet (2010, pp 7-9) refer to as “adversarial” – behaviour by one or two other members. However, many were motivated by the importance of the overall objectives. Volunteering and taking action in this way appears to have led to feelings of comfort in the uncertain environment of the pandemic.

## 5. Discussion and conclusion

### 5.1 Discussion

The results of the present study are consistent with the three main constructs of the radical collaboration framework:

**Inclusiveness.** Participants did report some signs of alienation, in having to join work that was already up and running, and regarding their own levels of expertise. In general, though, they reported a strong sense of sharing a common and globally significant role. The level of engagement and commitment appears to coincide with a perception that doing the right thing provides a stronger drive to overlook any such concerns.

**Distributed digital practices.** Tamm & Luyet (2010) stress both organisational and individual benefits associated with reducing conflict and perceptions of adversarial behaviours in the workplace. Participants were sensitive to and occasionally criticised issues of organisation, especially scheduling across multiple time zones. However, this did not undermine the commitment to the endeavour. Instead, there was significant engagement from the participants leading to an acceptance of minor adversarial behaviours and conflicts externally introduced through scheduling. Participants were able to make compromises, a sign that defensiveness was not a major issue.

**Productive and sustainable collaboration.** Participants reported a great sense of pride in what was achieved notwithstanding organisational limitations. They welcomed and appreciated the opportunity to work with others from different fields with whom they would not otherwise have had the opportunity to meet. Participants highlighted that their own confidence and skill levels had benefited from the experience.

Radical collaboration appears to describe the RDA COVID-19 WG initiative well. The motivation for engagement, though, may lie elsewhere. Overall, there was a sense of common purpose enabled by inclusivity and *Ingroup* identification. There were stronger motivators than constraints, and participation was empowering. Volunteers felt pride in the quality and importance of the work accomplished and acknowledged that it gave them a sense of control that mitigated the negative feelings imposed by the pandemic.

McGovern (2018a) described the digital environment in terms of a contemporary collaborative tool allowing geographically dispersed experts to engage with one another. Although Turkle described the short-term benefit of the virtual world, she argued that it ultimately leads to an increased sense of physical isolation ( Turkle, 2017). Social psychologists have similarly identified the socially disturbing effects of lockdowns and social distancing ( Jetten *et al.*, 2020). It is possible, however, that the challenges imposed by the distributed environment and lack of organisation were ignored given the benefit of inclusiveness. Participants engaged to make a difference during a crisis, but also to connect with similar others (their global *Ingroup*). It may be that the constructs of radical collaboration do not have equal importance. Inclusiveness may have been the primary (intrinsic) motivator which led to productive and sustainable collaboration. Willingness to compromise, trust inspired by the previous work of the RDA, and working alongside experienced RDA members allowed participants to exploit this collaborative experience for longer lasting personal benefit.

In summary, although the constructs of radical collaboration ( McGovern, 2018a; Tamm & Luyet, 2010) are well attested by respondents from the RDA COVID-19 WG, other factors such as intrinsic and extrinsic motivators as part of self-determination during the global health emergency appear to be significant.

### 5.2 Conclusion

The present study found that inclusiveness, distributed working, and productive and sustainable collaboration could usefully account for how contributing volunteers perceived the efforts to create the RDA data sharing recommendations. However, it became apparent that there were other factors such as motivation and the response to the disconnectedness as the result of the pandemic which may have influenced the success of collaboration under these extreme circumstances.

## 6. Limitations and future work

**1.    The study was based on a non-representative sample.**
  Survey questionnaire respondents were self-selecting and included co-authors of the present paper. Such potentially confounding factors should be controlled for in any future work ( Howards *et al.*, 2012; Weinberg, 1993).
**2.    Separation of any causal relationship between radical collaboration as an account of the external context and social factors in encouraging and maintaining engagement was not validated.**
  We also found evidence of strong social forces, such as the need to belong, the need to respond to an otherwise disorientating situation, and a sense of pride for the societal benefit associated with the final outcome. These are recognised characteristics of social identity/self-categorisation theories ( Hornsey, 2008). When an individual is aware of characteristics of a target group that they aspire to, they may respond by adopting those characteristics to assert membership and strengthen social ties ( Postmes *et al.*, 1999). The importance of social identification has also been discussed as a significant factor during the pandemic ( Jetten *et al.*, 2020).
  Separating any causal relationship between radical collaboration as an account of the external context and social factors in encouraging and maintaining engagement needs validation either empirically or via a meta-analysis of existing research especially during the pandemic. Possible models for an empirical investigation may be found in the health behaviour literature: the *Health Belief Model* and *Protection Motivation Theory* both identify the perception or response to risk as predictive of the intention to act, but also requiring self-efficacy through the adoption of that behaviour ( Conner & Norman, 2005). In this case, we would suggest that volunteers may have felt vulnerable because of the pandemic and of the unusual social constraints but saw the RDA initiative as an opportunity to take back some level of control and self-efficacy. Exploring the constructs of such models in future extreme collaborations may therefore provide further support for the relationship between external context and social identity. The threats from the context are therefore mitigated through engagement and collaboration with ingroup members.
**3.    The extent to which the flat organisational structure of the RDA embodied characteristics of an agile organisation was not explored. The role the organisational structure of the RDA played in enabling radical collaboration and agile development was not explored.**
  An analysis of the RDA organisational agility with respect to strategy, structure, process, people, and technology, would provide a more complete picture of enabling factors.
**4.    Europe and the Anglo-Saxon (Western) world were over-represented in the survey.**
  The sample from the present study was not comprehensive enough with non-Western cultures to be able to make any statement about potential cultural differences ( Wagner, 1995).
**5.    The role of the predominant language being English was not investigated.**
  A language barrier may be a factor which mitigates against inclusiveness and might encourage defensiveness (in not having the linguistic capacities that native speakers would have). However, these are largely academics, accustomed to communicating in English.
**6.    There was no comparison group such as, for example, the overall RDA membership or other RDA WG’s.**

## 7. Recommendations

The following recommendations are directed for consideration by the Research Data Alliance. Other similar organisations may also find them useful.

**1.   Encourage inclusiveness**
  Inclusiveness is one of the main constructs of radical collaboration. It is also a consequence of ingroup identification. One way to achieve this is to provide a social context within which participants can understand one another and each other’s discipline outside the specific goal of the collaboration. Therefore, collaborative efforts like the RDA COVID-19 WG should include non-task-oriented discussion and exchanges to help participants identify with collaborators and understand their motivators and ways of working.
**2.   Encourage intrinsic motivation**
  Collaborative efforts should seek to appeal to individual integrity and desire to contribute. A regular overview of what has been achieved and how individual groups have contributed may help.
**3.   Encourage sustainable engagement**
  - Organise regular checkpoints where individuals can monitor what is being achieved by the whole group; and
  - Encourage individuals to identify what they have gained as milestones are met.
**4.   Develop clear authorship and co-authorship guidelines for use by all RDA WGs and sub-WGs.**
  - Consider adapting the Open Research Europe publishing policy, or similar, for use by the RDA ( European Commission, 2021).
**5.   Explore causal relationships**
  between environment (such as the radical collaboration framework), social motivators (such as the need to belong and contribute) and the ultimate success of extreme collaborative initiatives.
**6.   Conduct a larger questionnaire-based survey across all RDA WGs to explore common and unique factors across groups, and to confirm the findings of the present study.**

## Data availability

### Underlying data

Digital Repository of Ireland ^2^: RDA COVID19 Community participation survey anonymised dataset

https://doi.org/10.7486/DRI.s752m1966 ( David *et al.*, 2021a)

This project contains the following underlying data:

- RDA_COVID19_Community_Participation_Survey_dataset_anonymised_November2020.xlsx
  Description: This is the fully anonymised version of the survey data associated with the present paper in a freely accessible Excel™ file.

Data are available under the terms of the Creative Commons Attribution 4.0 International license (CC-BY 4.0).

Digital Repository of Ireland: RDA COVID19 Community participation survey dataset

https://doi.org/10.7486/DRI.s178j828s ( David *et al.*, 2021b)

This project contains the following underlying data:

- RDA_COVID19_Community_Participation_Survey_dataset_pseudonymised_November2020.xlsx
  Description: This is a pseudonymised version of the survey data. Since pseudonymised data are considered personal data, access is restricted.

Access can be requested via the repository contact function. All requests will be forwarded to Romain David and Timea Biro who will liaise with co-authors to assess these on a case-by-case basis. The dataset will only be shared if requests align with the purpose, research objectives, and methods of the survey, and if there is a demonstrated commitment to protect the privacy of survey participants. This data object is not licensed for general reuse.

### Extended data

Digital Repository of Ireland: RDA COVID19 Community participation survey questionnaire blank

https://doi.org/10.7486/DRI.wd37kj848 ( David *et al.*, 2021c)

This project contains the following underlying data:

- RDA_COVID19_Community_Participation_Questionnaire.pdf
  Description: This is a blank copy of the survey questionnaire.

Data are available under the terms of the Creative Commons Attribution 4.0 International license (CC-BY 4.0).
