# Supplementary material for: Radical collaboration during a global health emergency: development of the RDA COVID-19 data sharing recommendations and guidelines
Source: Open Res Eur. 2021 Jun 16;1:69. [Version 1] doi: 10.12688/openreseurope.13369.1 (PMC10446077; doi:10.12688/openreseurope.13369.1)
Supplement: Peer review history

# Peer review: Reviewer response for version 1

**DOI:** 10.21956/openreseurope.14439.r27169

**Published:** 2022-04-11

**Authors:** Nancy McGovern

**Affiliations:** 1 Digital Preservation, MIT Libraries, Massachusetts Institute of Technology, Cambridge, MA, USA

**Article Overview**

 The paper presents the results of a study by the Research Data Alliance (RDA) of how RDA engaged researchers in developing their COVID 19 guidelines for data sharing. The authors established the need for more intentional collaboration during the pandemic to engage in research, both the organizational scaffolding and the researcher engagement needed, and utilized constructs from the radical collaboration model as introduced in the Research Library Issues volume 296 (Ruttenberg & Waraksa, 2018) including the introduction of the radical collaboration concepts and model (McGovern 2018) for their study. The paper includes an extensive analysis of the results using mixed methods. The analysis informed recommendations provided by the authors for collaborating across domains and geography for RDA and for use by other organizations. This report addresses the validity of the research presented in the paper.

**Introduction and theoretical background**

 The paper grounds the study in relevant literature while establishing the need for the study and providing a necessary description of the complex organization of RDA’s COVID 19 Working Group to enable readers to understand the study. The paper is well-written and presents the research clearly.

**Methods**

 The design for the study is novel as well as sound. A survey was an effective even obvious means for engaging working group members; the adaptation of collaborative model to develop the instrument was innovative. The mixed-methods used in the extensive analysis of the results lead to the extension of the model used for the study.

**Results**

 The extensive analysis presents sometimes complex results clearly with an effective sequence of text-based descriptions and data visualizations. This section is engaging and readable. The explanation of the methodology and the analysis provide more than sufficient information to enable replication. The underlying source data and materials are available.

**Discussions and conclusion**

 The quantitative and qualitative analysis and its interpretation are suited to the problem, clearly presented, and clearly supported by the results. The discussion and conclusion are sufficient if noticeably more concise than other sections of the paper. The authors demonstrate the utility of the model they adopted for their study and highlight the additional factors their study identified that enhance the original model.

**Limitations**

 The authors detail the limitations of the study in a way that is informative for reviewing this study and to inform researchers who might replicate or adapt their work.

**Recommendations**

 The recommendations identify possible next steps for the research that RDA might take and share lessons learned to benefit RDA’s future collaborative work.

If applicable, is the statistical analysis and its interpretation appropriate?

Yes

Is the study design appropriate and is the work technically sound?

Yes

Is the work clearly and accurately presented and does it engage with the current literature?

Yes

Are the conclusions drawn adequately supported by the results?

Yes

Are sufficient details of methods and analysis provided to allow replication by others?

Yes

Are all the source data and materials underlying the results available?

Yes

Reviewer Expertise:

My areas research are capacity building for lifecycle management within organizations, standards and good practice for digital preservation management, developing sustainable organizational programs, and radical collaboration, and inclusion frameworks.

I confirm that I have read this submission and believe that I have an appropriate level of expertise to confirm that it is of an acceptable scientific standard.

# Peer review: Reviewer response for version 1

**DOI:** 10.21956/openreseurope.14439.r27328

**Published:** 2022-03-28

**Authors:** Irene Pasquetto

**Affiliations:** 1 School of Information, U Michigan, Ann Arbor, MI, USA

Overall, the article presents novel and useful results that can surely guide future large-scale, distributed collaborative efforts in data sharing. The design of the study is well-constructed. The best part of the paper is the findings section, which is carefully detailed and contains several interesting results.

 Following the publication’s guidelines (i.e., “Reviewers are asked to assess the validity of the article content, rather than the novelty or interest levels”), I will now review the article in terms of its validity as a research article specifically, rather than the overall quality of its content.

 In its current format, the article lacks a major component of a research article, which is the engagement with the literature in the discussion section. The discussion reads like a short summary of results and it is only a half of a page long, which is quite unusual in social science. Authors should use the discussion section to explain how their findings are advancing our knowledge and understanding of radical collaborations in data sharing, compared to what was previously known before this study was conducted.

 Authors should consider re-scoping the article in terms of geographical participation and impact. I’m particularly concerned with the use of the term “global” to characterize an effort led by (or requested by) a European institution (EC). Also, the article should include in the lit. review an overview of other major, similar efforts conducted in the US and around the world, and discuss the RDA effort in relation to such efforts in the discussion.

 Sometimes, the authors use vague language and make claims without proper citations. For example, at page 4, the authors note that “The global community’s response was punctual, …”.  Which global community? RDA? “Punctual” is a value statement. Please re-phrase and use neutral language such as “relatively fast compared to...”. This is just an example of editorializing, the article should be edited as a whole to reduce editorializing and replace it with empirical, neutral language (or, if authors use value statements, they should provide evidence that supports such claims).

If applicable, is the statistical analysis and its interpretation appropriate?

Partly

Is the study design appropriate and is the work technically sound?

Yes

Is the work clearly and accurately presented and does it engage with the current literature?

Partly

Are the conclusions drawn adequately supported by the results?

Partly

Are sufficient details of methods and analysis provided to allow replication by others?

Yes

Are all the source data and materials underlying the results available?

Partly

Reviewer Expertise:

Open science and data sharing practices.

I confirm that I have read this submission and believe that I have an appropriate level of expertise to confirm that it is of an acceptable scientific standard, however I have significant reservations, as outlined above.

# Peer review: Reviewer response for version 1

**DOI:** 10.21956/openreseurope.14439.r28217

**Published:** 2022-01-19

**Authors:** James Scheibner

**Affiliations:** 1 Government and Law,, Flinders University, Adelaide, Australia

**Article overview**

 The following article focuses on the efforts of the Research Data Alliance (RDA) to develop guidelines for COVID related data sharing. Specifically, the article seeks to determine how the RDA succeeded in engaging scientists to help develop these guidelines. The authors attempt to measure this engagement through a survey that was distributed to RDA COVID-19 workgroup members.

**Background**

 Sections 1 (Introduction) and 2 (Theoretical Background) describe the background underpinning this paper. The literature review is sound and does not require any amendment.

**Methodology**

 The authors received approval from an ethics commitment (see section 3.1). The survey described in this article was based on three main indicators of radical collaboration as defined by McGovern, 2018. These indicators are inclusive community, distributed digital practices, and productive and sustainable collaboration. Each dimension was measured against the experiences of RDA workgroup members via a series of Likert scale responses and open-ended questions. The authors piloted this questionnaire prior to distributing it to members of the RDA group (see section 3.3). The authors then downloaded the quantitative results from the Likert scale analysis into a CSV file. The qualitative results with free-form text were downloaded into a word document. The authors extracted quotes using qualitative data analysis software. Finally, the authors anonymised the pseudonymised data using sdcMicro before making it publicly available. All of these processes described by the authors represent practice for qualitative survey methods.

 The only point of concern relates to the level of detail regarding data collection in section 3.3. Some more detail, such as how long they waited for results from the research participants and when the survey was distributed would help add to the analysis.

**Results**

 The authors clearly state how many of their respondents were included in their analysis, as well as the demographics of their respondents (see section 3.4). The authors then went on to assess the qualitative aspects of the three main indicators of radical collaboration. In particular, the authors used excerpts from surveys and diagrams effectively to demonstrate how respondents positively and negatively described the RDA COVID-19 working group as a collaborative forum (see figures 12, 13, 14, 16 and 21). I am not personally qualified to comment on the statistical robustness of the survey. However, the authors have recognised this in their limitations section. From a qualitative perspective the underlying analysis is a particularly strong mixed methods approach.

**Discussion and Conclusion**

 The authors note that their findings are consistent with McGovern's radical collaboration framework. In particular, the authors note that inclusiveness may act as an incentive to collaborate overriding the challenges imposed by working remotely. However, the authors also note that other factors such as the disconnectedness as a result of the pandemic may have acted as a motivation. This discussion and conclusion are derived from the results that are derived and are carefully considered.

**Limitations**

 The authors addressed some of the key limitations of their study. These include the fact that the survey was not represented, European respondents were overrepresented and the impact of English being the dominant language on inclusiveness was not measured. The authors also recognise that there might be not a causal relationship between radical collaboration and the factors in maintaining engagement. The lack of causality is a potential weakness of qualitative and mixed methods approaches, and it is important for the authors to have recognised this.

**Recommendations**

 The recommendations proposed by the authors are derived from the conclusions of the article, and establish the groundwork for further research.

**Summary Peer Review**

 Overall, this article is very impressive and clearly applies a solid mixed methods framework to radical remote collaboration during the COVID-19 pandemic. My only recommendation is slightly more detail about the data collection phase.

If applicable, is the statistical analysis and its interpretation appropriate?

Yes

Is the study design appropriate and is the work technically sound?

Yes

Is the work clearly and accurately presented and does it engage with the current literature?

Yes

Are the conclusions drawn adequately supported by the results?

Yes

Are sufficient details of methods and analysis provided to allow replication by others?

Yes

Are all the source data and materials underlying the results available?

Yes

Reviewer Expertise:

My areas of research are Patent Law, Copyright Law, Open Source Law, Data Privacy, Health Law and Policy, Bioethics, Mixed Methods Research, Qualitative Research and Institutional Economics. Note that I am not qualified to conduct an analysis of the statistical integrity of the results.

I confirm that I have read this submission and believe that I have an appropriate level of expertise to confirm that it is of an acceptable scientific standard.
